# Human mutations in integrator complex subunits link transcriptome integrity to brain development

**DOI:** 10.1371/journal.pgen.1006809

**Published:** 2017-05-25

**Authors:** Renske Oegema, David Baillat, Rachel Schot, Leontine M. van Unen, Alice Brooks, Sima Kheradmand Kia, A. Jeannette M. Hoogeboom, Zheng Xia, Wei Li, Matteo Cesaroni, Maarten H. Lequin, Marjon van Slegtenhorst, William B. Dobyns, Irenaeus F. M. de Coo, Frans W. Verheijen, Andreas Kremer, Peter J. van der Spek, Daphne Heijsman, Eric J. Wagner, Maarten Fornerod, Grazia M. S. Mancini

**Affiliations:** 1 Department of Clinical Genetics, Erasmus MC University Medical Center, Rotterdam, The Netherlands; 2 Department of Biochemistry & Molecular Biology, University of Texas Medical Branch, Galveston TX, United States of America; 3 Division of Biostatistics, Dan L Duncan Cancer Center, Baylor College of Medicine, Houston, TX, United States of America; 4 Department of Molecular and Cellular Biology, Baylor College of Medicine, Houston, TX, United States of America; 5 The Fels Institute, Temple University School of Medicine, Philadelphia, PA, United States of America; 6 Department of Pediatric Radiology, Erasmus MC- Sophia, University Medical Center Rotterdam, The Netherlands; 7 Center for Integrative Brain Research, Seattle Children's Research Institute, Seattle, Washington, United States of America; 8 Department of Neurology, Erasmus MC- Sophia, University Medical Center Rotterdam, The Netherlands; 9 Department of Bioinformatics, Erasmus MC, University Medical Center Rotterdam, The Netherlands; 10 Department of Pediatric Oncology and Biochemistry, Erasmus MC, University Medical Center Rotterdam, The Netherlands; Stanford University School of Medicine, UNITED STATES

## Abstract

Integrator is an RNA polymerase II (RNAPII)-associated complex that was recently identified to have a broad role in both RNA processing and transcription regulation. Importantly, its role in human development and disease is so far largely unexplored. Here, we provide evidence that biallelic *Integrator Complex Subunit 1 (INTS1)* and *Subunit 8* (*INTS8)* gene mutations are associated with rare recessive human neurodevelopmental syndromes. Three unrelated individuals of Dutch ancestry showed the same homozygous truncating *INTS1* mutation. Three siblings harboured compound heterozygous *INTS8* mutations. Shared features by these six individuals are severe neurodevelopmental delay and a distinctive appearance. The *INTS8* family in addition presented with neuronal migration defects (periventricular nodular heterotopia). We show that the first *INTS8* mutation, a nine base-pair deletion, leads to a protein that disrupts INT complex stability, while the second missense mutation introduces an alternative splice site leading to an unstable messenger. Cells from patients with *INTS8* mutations show increased levels of unprocessed UsnRNA, compatible with the INT function in the 3’-end maturation of UsnRNA, and display significant disruptions in gene expression and RNA processing. Finally, the introduction of the *INTS8* deletion mutation in P19 cells using genome editing alters gene expression throughout the course of retinoic acid-induced neural differentiation. Altogether, our results confirm the essential role of Integrator to transcriptome integrity and point to the requirement of the Integrator complex in human brain development.

## Author summary

Neurodevelopmental disorders often have a genetic cause, however the genes and the underlying mechanisms that are involved are increasingly diverse, pointing to the complexity of brain development. For normal cell function and in general for normal development, mechanisms that regulate gene transcription into mRNA are of outermost importance as proper spatial and temporal expression of key developmentally regulated transcripts is essential. The Integrator complex was recently identified to have a broad role in both RNA processing and transcription regulation. This complex is assembled from at least 14 different subunits and several animal studies have pointed to an important role in development. Nevertheless, studies directly demonstrating the relevance of this complex in human health and development have been lacking until now. We show here that mutations in the *Integrator Complex Subunit 1 gene (INTS1)* and *Subunit 8* gene (*INTS8)* cause a severe neurodevelopmental syndrome, characterized by profound intellectual disability, epilepsy, spasticity, facial and limb dysmorphism and subtle structural brain abnormalities. While the role of the Integrator complex in neuronal migration has recently been established, we provide evidence that INTS8 mutations lead *in vitro* to instability of the complex and impaired function. In patients cultured fibroblasts we found evidence for abnormalities in mRNA transcription and processing. In addition, introduction of *INTS8* mutations in an *in vitro* model of retinoic acid-induced neuronal differentiation results also in transcription alterations. Altogether our results suggest an evolutionary conserved requirement of INTS1 and INTS8 in brain development.

## Introduction

Malformations of cortical development (MCD) are a group of neurodevelopmental disorders characterized by structural brain abnormalities involving the human cerebral cortex. They form a common cause of developmental delay and epilepsy, accounting for 3% of intellectual disability, 25% of pediatric partial seizures, 5–15% of adult epilepsy, and 20–40% of therapy-resistant epilepsy [1–5]. MCD are divided into three main groups, reflecting failure of either the neurodevelopmental process of cell proliferation, neuronal migration, or post migrational cortical organization [6]. It was anticipated that mutations in genes unique to each disorder and to specific developmental stages would be identified. Recently, this classification has been challenged by the discovery of mutations in genes like *WDR62* and *TUBA1A* [1, 7, 8] that lead to multiple malformations originating in different stages of brain development. Among MCDs, periventricular nodular heterotopia is considered a developmental defect of neuronal progenitors that fail to migrate from the ventricular and subventricular zone toward the upper cortical layers during early embryogenesis or undergo premature differentiation in apparent neuronal lineage. The most common genetic cause of periventricular nodular heterotopia is a mutation in the *FLNA* gene, which is associated with normal cognitive development. Rare gene mutations including ARFGEF2 and several rare chromosomal aberrations explain a small proportion of syndromic periventricular nodular heterotopia, associated with other malformations and/or developmental delay (for review see [6, 9]). It is rarely associated with cerebellar hypoplasia and microcephaly. Moreover, many cases of periventricular heterotopia remain without molecular etiology [10].

The Integrator complex (INT) consists of at least 14 subunits and is phylogenetically conserved among metazoans [11, 12]. Integrator associates with the C-terminal domain of the largest subunit of the RNA polymerase II (RNAPII) and has a role in the regulation of gene expression and RNA processing. It was first discovered to mediate the co-transcriptional 3’-end processing of the U-rich small nuclear RNAs (UsnRNAs), the RNA components of the spliceosome [11, 13]. Recently, the scope of Integrator complex function has broadened through the discoveries of a general role in RNAPII promoter proximal pause-release and in the processing of enhancer RNAs [14–18]. Most Integrator complex subunits bear no homology to any RNA processing machinery or transcriptional regulators that would allow a predictable function within the complex, with the exception of Integrator complex subunits INTS9 and INTS11 [19]. These subunits are paralogues of the cleavage and polyadenylation specificity factor subunits CPSF100 and CPSF73 respectively, which form the endonuclease factor responsible for cleavage of pre-mRNA at the polyA site [20–22]. Although animal studies suggest an evolutionary conserved requirement of Integrator complex subunits for normal embryonic development [23–28], human germline mutations have not yet been linked to disease.

Interestingly, mutations in UsnRNA have been previously linked to splicing alterations leading to brain disorders. For example, mutations in the mouse *Rnu2-8*, coding for U2 snRNA, result in cerebellar degeneration [29], *RNU4atac* mutations in humans result in extreme microcephaly [30] and mutations in *RNU12* have been found associated with early-onset cerebellar ataxia[31]. Recently, mutations in the non-canonical deadenylase TOE1 that trims the 3’ ends of pre-UsnRNA, have been linked to pontocerebellar hypoplasia[32]. Moreover, promoter-proximal pausing, affecting up to 40% of the genes, is particularly important during development [33, 34]. Paused RNAPII is preferentially encountered at developmentally-regulated genes where it can orchestrate the synchronous gene activation necessary for pattern formation [35–38]. This process is particularly important during neuronal development, synapse plasticity and maturation [39–41]. Altogether, these data indicate that, through UsnRNA processing and RNAPII promoter-proximal pausing, reduced Integrator activity could have an impact on normal human neurodevelopment.

Here, we describe the first report of INT mutations that are associated with severe neurodevelopmental defects. We identified mutations in two distinct INT subunits, INTS1 and INTS8, which are present in six patients from four unrelated families. We determined that patients with *INTS8* mutations carry two distinct alleles where one leads to the production of an unstable transcript while the other deletes three conserved amino acids near the C-terminus that disrupts integrity of the whole complex. We detect significant splicing and transcriptional defects in patients cells and demonstrate that replacement of intact *INTS8* genes with the deletion mutant using genome editing is sufficient to disrupt retinoic-acid induced neuronal differentiation in P19 cells.

## Results

### Biallelic *INTS1* and *INTS8* mutations

**Fig 1:**
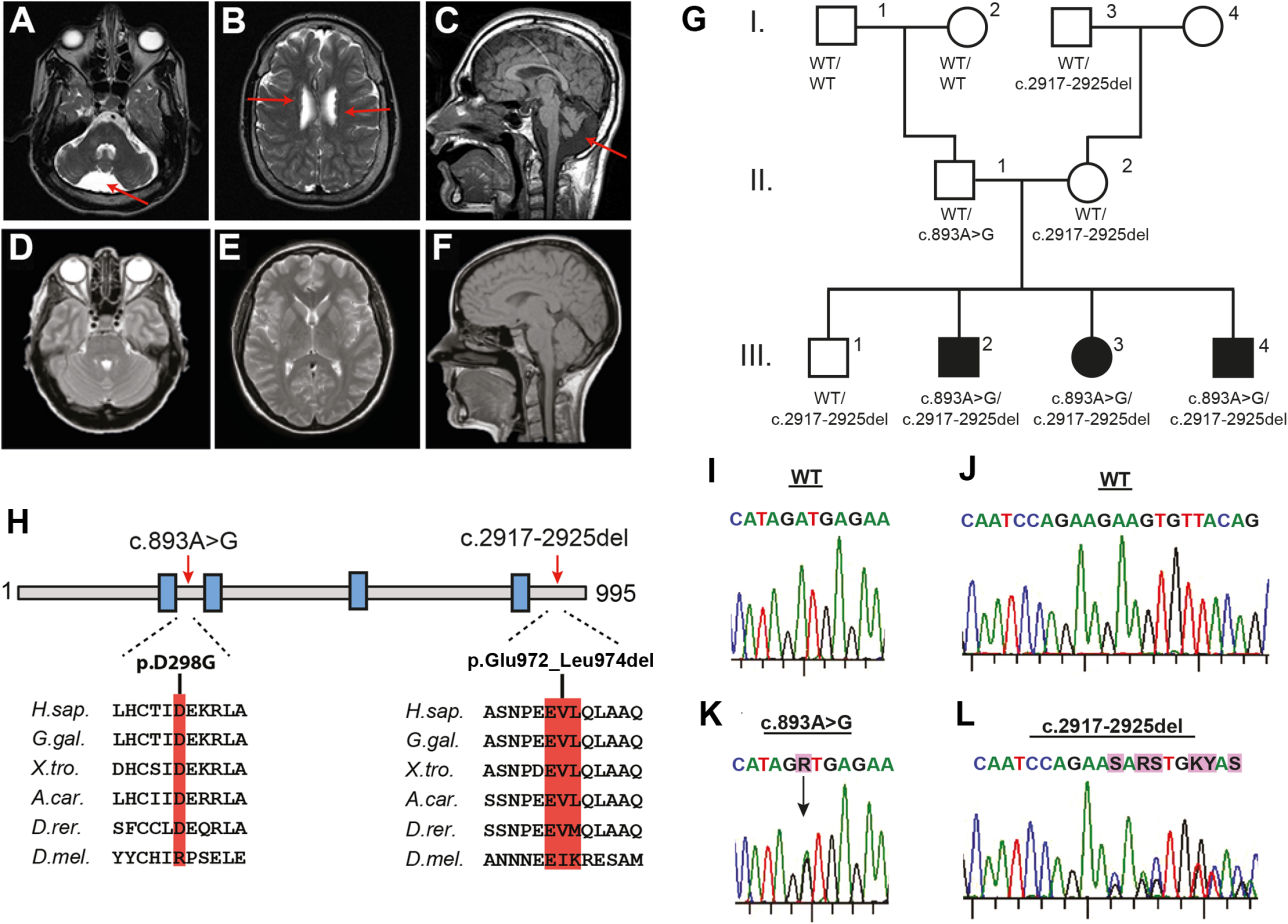
Biallelic *INTS8* mutations in a family with a severe neurodevelopmental syndrome. (A-C) Magnetic resonance imaging (MRI) of affected individual III-2 showing cerebellar hypoplasia (A,C, arrow), reduced volume of the pons and brainstem and periventricular nodular heterotopia (B, arrows) versus (D-F) normal MRI from unaffected individual. (G) Pedigree of the extended family; filled symbols represent affected individuals. Below each individual the *INTS8* alleles (wt = wild type) are shown. (H) Schematic of INTS8 including the four tetratricopeptide (TPR) motifs (blue blocks), the patient mutations and in the lower panel the conservation of the affected amino acids residues throughout evolution. (I-L) Electropherograms from Sanger sequencing of *INTS8* wild type and mutant alleles.

In our research cohort of patients with brain abnormalities we identified six individuals from four families with a distinct and recognizable neurodevelopmental syndrome. Clinical details are presented in Table 1. In summary, all patients shared profound intellectual disability, lack of speech development, motor impairment, seizures and similar dysmorphic features of the face and limbs. In addition, the three individuals from the same sibship had severe spastic tetraplegia, borderline microcephaly, and an abnormality at brain MRI: cerebellar hypoplasia, reduced volume of pons and brainstem and periventricular nodular heterotopia (PNH), a migration defect of the cortical neurons (Fig 1A–1F).

**Table 1:** Clinical phenotype of human *INTS1* and *INTS8* mutations.

	*INTS8* patient 1	*INTS8* patient 2	*INTS8* patient 3	*INTS1* patient 1	*INTS1* patient 2	*INTS1* patient 3
**Type of mutation**	c.893A>G, p.Asp298Gly; c.2917_2925del, p.Glu972_Leu974del	c.893A>G, p.Asp298Gly; c.2917_2925del, p.Glu972_Leu974del	c.893A>G,p.Asp298Gly; c.2917_2925del, p.Glu972_Leu974del	c.5351C>A, p.(Ser1784*), homozygote	c.5351C>A, p.(Ser1784*), homozygote	c.5351C>A, p.(Ser1784*), homozygote
**Age at last observation**	35 yr	32 yr	30yr	9 yr	19 yr	6 yr
**Gender**	male	female	male	female	male	male
**Growth parameters**						
stature	nd	-2 SD	-2 SD	-3 SD	- 4 SD	- 3 SD
head circumference	-3 SD	-2 SD	-2.5 SD	0 SD	0 SD	0 SD
**Neurology**						
Cognitive delay	severe	severe	severe	Moderate/severe	severe	severe
Language development	no	no	no	no	no	no
Motor impairment	Non ambulant, spastic paraplegia	Non ambulant, spastic paraplegia	Non ambulant, spastic paraplegia	Walks with aid	Walks with aid	Non ambulant
Seizures	yes	yes	yes	yes	no	no
Brain MRI scan	Cerebellar hypoplasia, Nodular heterotopia	Cerebellar hypoplasia, nodular heterotopia	Cerebellar hypoplasia, nodular heterotopia	Hypoplastic cerebellar inferior vermis	Normal	normal
**Congenital abnormalities**						
Face	Prominent glabella	Prominent glabella, hypertelorism	Prominent glabella, hypertelorism	Frontal bossing, hypertelorism, dolicocephaly	Frontal bossing, hypertelorism, dolicocephaly, cleft lip and palate	Frontal bossing, hypertelorism, dolicocephaly
Skeletal/Limbs	Irregularly implanted and overlapping toes	Irregularly implanted and overlapping toes	Irregularly implanted and overlapping toes	Pectus abnormality, Irregularly implanted and overlapping toes	Pectus abnormality, Irregularly implanted and overlapping toes	Pectus abnormality, Irregularly implanted and overlapping toes
Visceral organs	normal	normal	normal	normal	Renal dysplasia	Congenital heart defect
**Vision**	No visual contact	Optic atrophy	Optic atrophy	cataract	cataract	cataract

Two of the other unrelated individuals of Dutch ancestry sharing a similar phenotype underwent exome sequencing for clinical diagnostic purposes. In both patients the same homozygous nonsense mutation in *INTS1* was found c.5351C>A, p.(Ser1784*), (NM_001080453), whereas all the parents were heterozygote for the mutation. The last individual of Dutch ancestry was diagnosed because of recognizable dysmorphic features and was found by targeted analysis to be homozygote for the p.(Ser1784*) mutation, while his parents were heterozygotes. Additional genomic analysis, using Illumina Infinium_CytoSNP_850K v1.1 genotyping array, was performed which showed in all three individuals a shared region of homozygosity (according to UCSC Genome Browser Mar. 2009 (NCBI37/hg19)), suggesting this mutation was derived from a common ancestor. This mutation is not reported in ExAC (http://exac.broadinstitute.org), nor in gnomAD databases (http://gnomad.broadinstitute.org/). Expression of mRNA bearing the p.Ser1784* mutation in skin fibroblasts derived from two patients was significantly reduced when tested by qRT-PCR (S1 Fig), compatible with a loss of function effect.

Whole genome sequencing (WGS) using DNA from the three affected siblings and three unaffected family members identified biallelic mutations in the *Integrator Complex subunit 8* gene (*INTS8*) in the affected individuals, following an autosomal recessive inheritance pattern (Fig 1G, S1 and S2 Tables). The first mutation is a predicted missense mutation (c.893A>G, p.Asp298Gly), and the second is an in-frame nine-base-pair deletion leading to the deletion of three amino acids (c.2917_2925del, p.Glu972_Leu974del; simplified as INTS8ΔEVL in the rest of the text) (Fig 1H). Both mutations are not reported in either the ExAC or the gnomAD databases. The mutations and their segregation in the family were confirmed by Sanger sequencing (Fig 1I–1L). Interestingly, the c.893A>G mutation arose *de novo* in the father. INTS8 is a 995-amino-acid protein, containing four predicted tetratricopeptide (TPR) motifs, which are versatile protein-protein interaction domains. Both mutations reside in conserved regions of the protein and INTS8ΔEVL is located at the C-terminus of the TPR4 domain in a predicted alpha helix (Fig 1H). In spite of sequencing *INTS8* in 25 PNH patients and 266 other patients with brain malformations, we did not observe additional biallelic *INTS8* mutations, which suggests that *INTS8* mutational rate is very low. This is supported by the high combined annotation dependent depletion (CADD) score of the mutations (S3 Table) [42] and, with a score of 0.99, being classified as an extremely “loss-of-function intolerant” gene in the ExAC database (http://exac.broadinstitute.org).

### INTS8 c.893A>G mutation

**Fig 2:**
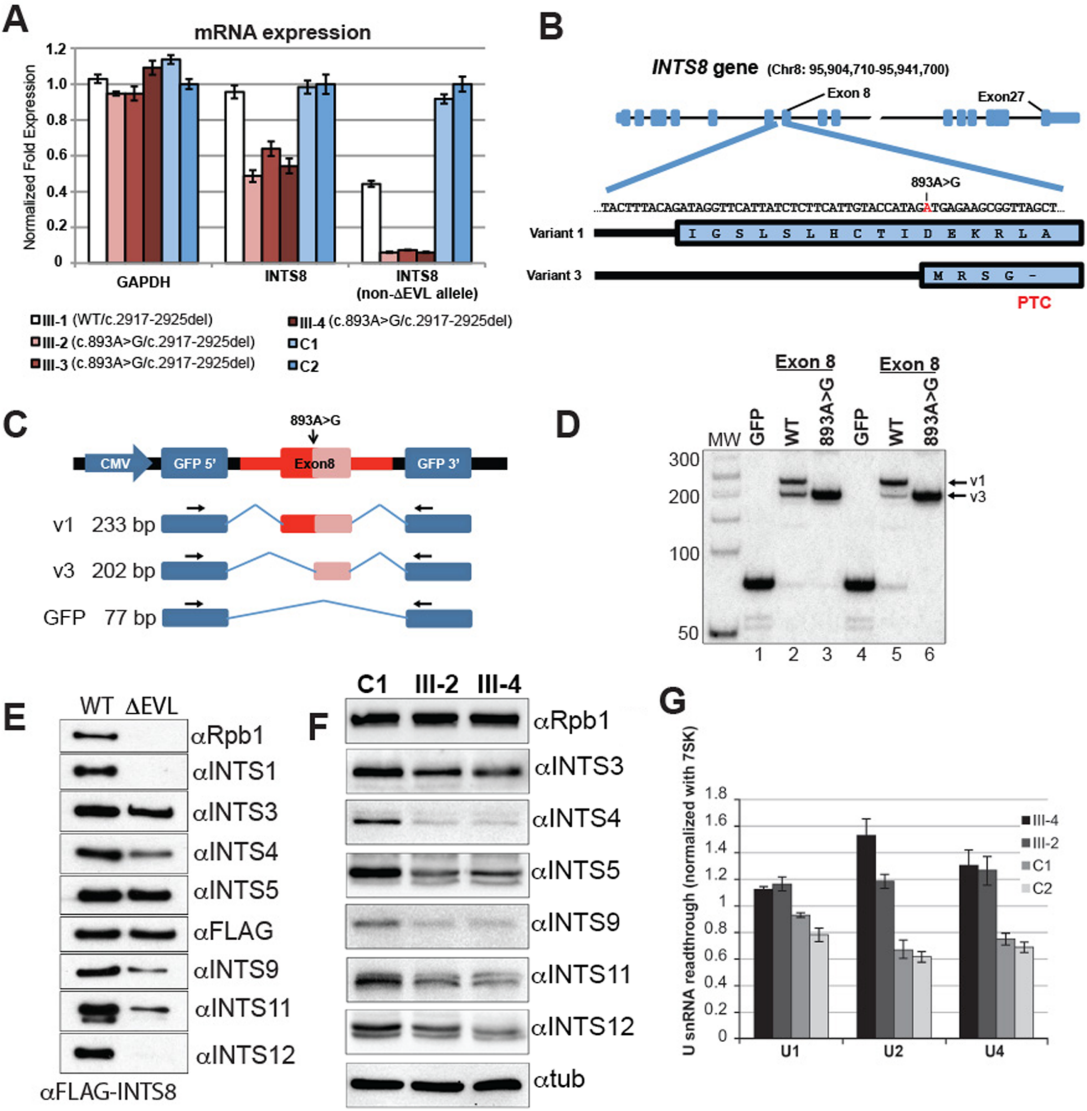
Characterization of the *INTS8* mutations. (A) qRT-PCR on fibroblast-derived RNA of the patients (III-2, III-3, III-4), their unaffected sibling (III-1), and two age-matched control cell lines (C1, C2), normalized for *GAPDH* expression. Expression of the c.893A>G allele vs. wild type was measured using a primer located at the c.2917-2925del locus (*INTS8* non-ΔEVL allele). (B) Schematic overview of INTS8 genomic and protein sequence. The c.893A>G mutation (in red) is located at the 5’ end of the exon8 of the transcript variant 3 that contains a premature stop codon (PTC). (C) Schematic of the GFP-minigene reporter construct used to evaluate the effect of the c.893A>G mutation on *INTS8* exon 8 splicing pattern. Size of the corresponding amplicons is indicated on the left (D) RT-PCR analysis of RNA isolated from HeLa (lanes 1–3) or HEK293T cells (lanes 4–6) transfected with the GFP-minigene constructs. The empty reporter (GFP) is used as a control. (E) Western blot analysis of flag-affinity eluates from HEK293T stable lines expressing 3xFlag-tagged INTS8 wild type (WT) or INTS8ΔEVL. (F) Western blots on total cell extracts from patient and control primary fibroblasts. (G) qRT-PCR showing normalized expression of misprocessed U1, U2 and U4 snRNAs in total RNA extracted from patient III-2 and III-4 fibroblasts compared to two controls. All pairwise comparisons between patient and control UsnRNA levels are significant (at least p<0.05, Student’s T-test) to the exception of III-2 and C1 for UsnRNA U1 (p<0.06).

To study whether and how these mutations affect *INTS8* expression we cultured cell lines from primary fibroblasts from the three affected siblings and their unaffected brother, who is heterozygous only for the *INTSΔEVL* mutation. qRT-PCR analysis indicates that *INTS8* expression is reduced in patient cells (~50% reduction) suggesting that one of the mutant *INTS8* alleles is not expressed (Fig 2A). To test this possibility, we designed a different set of primers incapable of amplifying the *INTS8ΔEVL* allele and found that the expression of the *INTS8* c.893A>G allele is almost undetectable in patients (Fig 2A).

Close analysis of known *INTS8* transcripts in the NCBI RefSeq database indicates that the *INTS8* c.893A>G mutation is located at position +1 of the exon 8 of an annotated alternative 3′ splice site used in isoform NR_073445.1 (variant 3, Fig 2B). This variant is predicted to generate a premature stop codon within exon 8 and to be subjected to rapid degradation through nonsense mediated decay (NMD). To test the impact of c.893A>G on exon 8 splicing, we designed an *INTS8*-GFP minigene-reporter construct (Fig 2C). Wild-type-*INTS8* minigenes transfected into HeLa or HEK293T cells generated two distinct exon inclusion products (Fig 2D, lane 2 and 5), confirmed by Sanger sequencing to be variants 1 and 3 (S2A and S2B Fig). Strikingly, introduction of the single base c.893A>G mutation into the *INTS8* reporter was sufficient to change the splicing of exon 8 so that the distal 3′ splice site used in the *INTS8* variant 3 was now exclusively utilized (Fig 2D, lane 3 and 6). Cloning and sequencing of this single spliced product confirmed the predicted exon junction (S2C Fig). Both endogenous isoforms can be detected in HeLa cells but variant 3 constitutes only 6% of total cellular *INTS8* mRNA (S2D and S2E Fig). Treatment of HeLa or HEK293T cells with puromycin or cycloheximide increased variant 3 RNA levels to 43% and 34%, respectively (S2E Fig), which indicates that variant 3 is subject to rapid degradation, probably through NMD [43]. Similarly, baseline levels of *INTS8* variant 3 in patient cells are also increased after mRNA stabilization by puromycin or cycloheximide treatment (S2F Fig). Collectively, these results show that the c.893A>G mutation dramatically alters the splicing of *INTS8* exon 8, producing a premature stop codon and an unstable transcript.

### *INTS8* c.2917_2925del mutation

As the c.893A>G allele produces an unstable mRNA, we deduced that the mutant INTS8ΔEVL is the preponderant protein expressed as suggested by the levels of mRNA expression in patient cells (Fig 2A, middle panel). Therefore, we investigated whether the INTS8ΔEVL protein could impact INTS8 interaction with the other subunits in the complex. To that end, we established HEK293T cells stably expressing wild-type (WT) or mutant (ΔEVL) INTS8 bearing an N-terminal 3XFLAG tag and purified the associated complex using anti-FLAG affinity resin. WT and mutant INTS8-associated peptides showed a very similar pattern by SDS-PAGE (S3 Fig). Probing for the presence of specific Integrator complex subunits by western blot revealed however differential association with the two proteins. The FLAG-INTS8-ΔEVL eluates contained nearly undetectable amounts of INTS1,-12 and RPB1, reduced levels of associated INTS4, -9 and -11, but similar levels of INTS5 and INTS3, compared to FLAG-INTS8–WT (Fig 2E). Thus, the ΔEVL mutation appears to impact the ability of INTS8 to associate with selected members of the Integrator complex. In patient cells, we found slightly reduced levels of several INT subunits including INTS3, INTS5, INTS11 and INTS12 (Fig 2F). We also observed significantly reduced levels of INTS4 and INTS9 (Fig 2F). The reduction in quantity of several INT subunits could be due to reduced mRNA expression or protein accumulation suggesting that the reduced association of INTS8 with certain members of Integrator complex could lead to an overall loss in the protein complex integrity and affect in return the stability of some of its subunits. Consistent with this observation, we measured slight but significantly elevated amounts of misprocessed UsnRNA in patient cells (Fig 2G), while total UsnRNA levels did not significantly differ between patient and control cells (S4 Fig). Taken together, these results indicate that the *INTS8* mutations lead to both a reduction in integrity and function of the Integrator complex.

### Alteration of gene expression and splicing patterns genome wide

**Fig 3:**
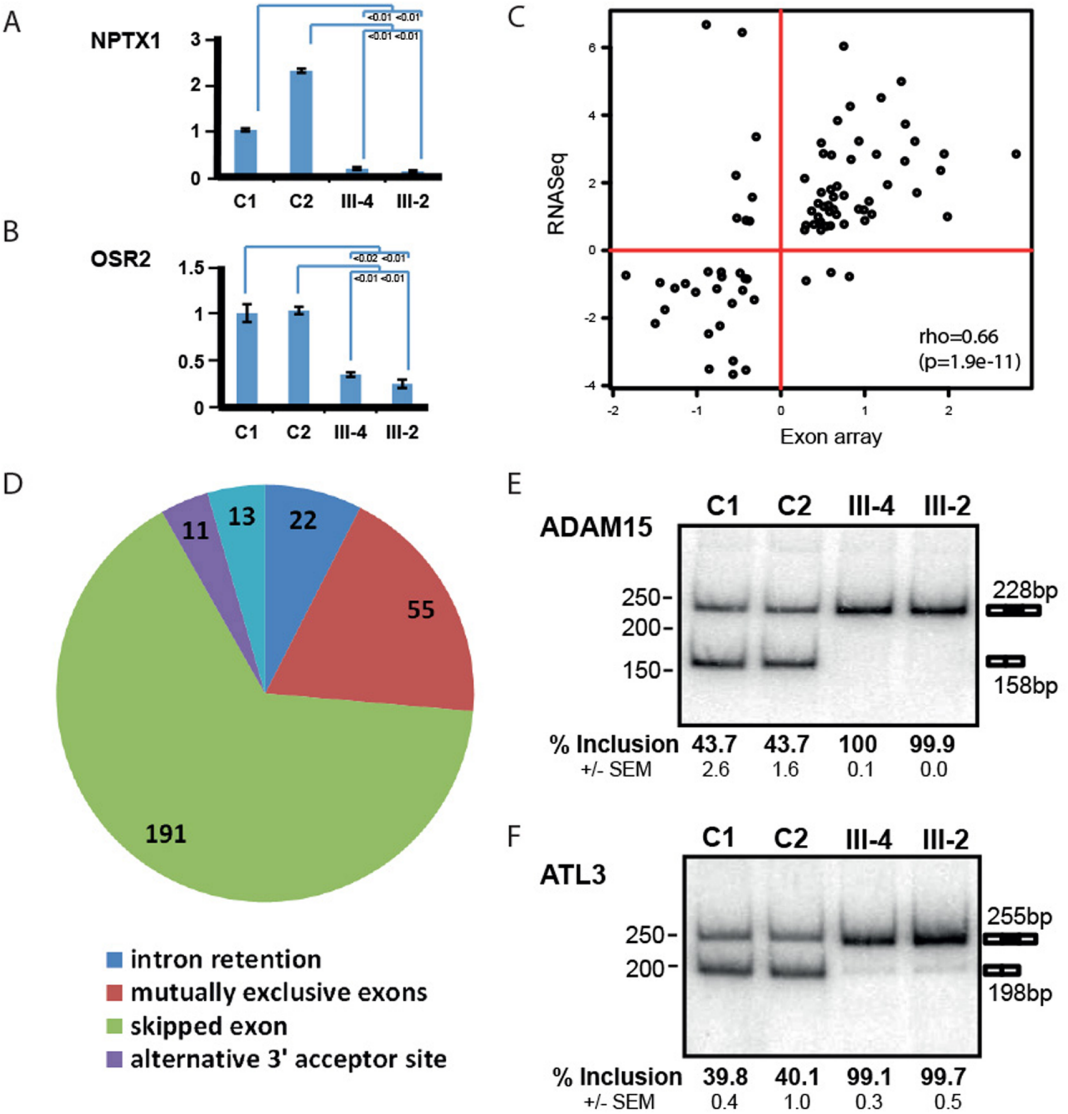
Dysregulated transcriptome in patient skin fibroblasts. **(**A, B) qRT-PCR validation of gene expression variation in patient cells for two illustrative examples, *NPTX1* and *OSR2* mRNAs. (C) Correlation analysis of differential gene expression data from exon arrays (X axis) and RNA-seq (Y axis). (D) Pie chart representing the different types of alternative splicing events detected in patient cells vs control in RNA-seq data (n = 215, p<0.01, 292 total events). (E, F) Experimental verification by RT-PCR of the splicing changes associated with *INTS8* mutations for two illustrative examples *ADAM15* (E) and *ATL3* (F) mRNAs.

To investigate the extent of *INTS8* dysfunction on transcription and splicing, we conducted both exon array analysis and RNA-seq on patient cells relative to controls. Differential gene expression (DGE) data from exon arrays performed on fibroblasts showed a large number of significantly dysregulated genes, including *INTS8*, in the patient vs. control cells (n = 682; p<0.02, S4 Table). To confirm both the results and the reproducibility attained by exon array we selected four of the highly dysregulated genes that are also known to be expressed in brain and important in CNS development and tested their expression using qRT-PCR. Importantly, we were able to successfully confirm the results using two independent qRT-PCR experiments comparing the three patients with two new and independent age-matched fibroblast control cell lines (S5 Fig). To further explore a genome-wide effect on splicing we subjected poly(A) mRNA fractions from patient III-2 and III-4 and 2 age-matched controls to high depth RNA-seq analysis (~65-fold average coverage, S5 Table). We found an even larger number of genes (N = 3,002; p<0.02) that were significantly up- or downregulated in patient cells vs. control cells (S6 Table). We validated 17 of these potential target genes by qRT-PCR (illustrative examples are in Fig 3A and 3B, S6 Fig). Comparison of DGE data between exon arrays and RNA-seq showed a correlation coefficient of 0.66 between the two data sets (Fig 3C) and, interestingly, that a majority of the genes that are significantly dysregulated in both datasets (N = 82) are expressed in the CNS (S7 Table).

In addition to transcriptional deregulation, we also detected significant splicing changes in 215 genes (p<0.01, 292 total events, S8 Table) and confirmed the *INTS8* alternative splicing induced by the c. 893A>G mutation in patient cells. The vast majority of affected splicing events are skipped exons (65%) and mutually exclusive exons (19%, Fig 3D). We selected four alternatively spliced genes and confirmed the corresponding splicing changes using RT-PCR assays with primers flanking differentially spliced regions (Fig 3E and 3F, S7 Fig). Altogether, these multiple analyses demonstrate broad changes in both splicing and transcript levels in patient cells containing disrupted *INTS8* expression.

### *INTS8* expression during mouse and human fetal brain development

In situ hybridization (ISH) data in mouse embryonic brain show a high expression of *INTS8* in the CNS at E14.5, especially in the brain cortex ventricular zone (VZ) and hindbrain (S8 Fig)[44]. In humans, heat maps extracted from expression array data of several brain areas show high expression of *INTS8* in the ventricular and subventricular zones, caudal and lateral ganglionic eminences and cerebellar primordium at 16–21 postconceptional week (pcw) (S8 Fig). In the first and second trimester, from the ganglionic eminences, GABAergic interneuron progenitors migrate tangentially in the subventricular and marginal zone of the telencephalon in order to organize cortical development; meanwhile the subventricular zone is also an area of active proliferation of glutamatergic neural progenitors [45]. Analysis with the R software of raw RNAseq data of *INTS8* expression at several developmental stages (range 8 pcw—40 years) across different human brain areas and multiple individuals, obtained from BrainSpan, shows that *INTS8* expression peaks during early embryonic development, and decreases to retain a stable level during postnatal life (S9 Fig). The spatiotemporal expression pattern of *INTS8* suggests co-localization with both pyramidal and interneuron progenitors regulating neuronal migration and is in accordance with the PNH phenotype.

### In vitro differentiation of INTS8ΔEVL mutant cell

**Fig 4:**
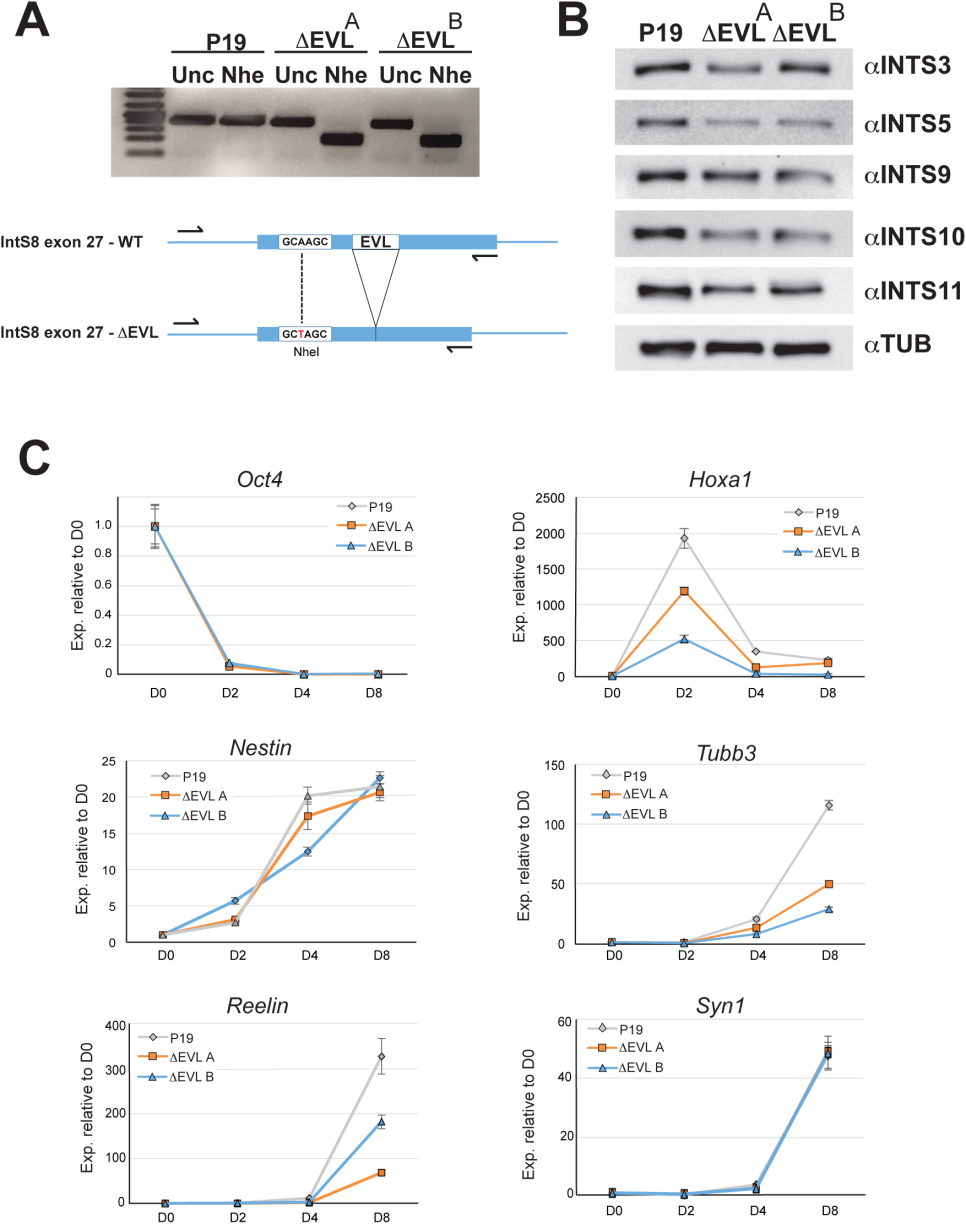
Effect of *INTS8ΔEVL* mutation on P19 cell neuronal differentiation. (A) Two P19 clonal cell lines bearing a homozygous *INTS8ΔEVL* mutation were generated by CRISPR/Cas9 mediated genome editing using two different guide RNAs. After genomic DNA extraction, the region surrounding the *INTS8ΔEVL* mutation is amplified by PCR and the corresponding DNA digested with NheI to detect homologous recombination or mock digested (Unc = uncut). The P19 parental cell line is used as a control. (B) The protein expression of different INT subunits is monitored by Western Blot in total cellular extracts of *INTS8ΔEVL* mutant P19 cell lines. The P19 parental cell line is used as a control. Tubulin serves as a loading control. (C) Expression of neuronal differentiation markers during RA-induced differentiation of P19 cells. Wild-type and *INTS8ΔEVL* P19 cell lines are treated with RA and let to differentiate for 8 days. Cells are harvested at the indicated time points after the initiation of the differentiation protocol (D0 = day zero, D2 = day two, D4 = day4, D8 = day8) and RNA was extracted and reverse transcribed. Gene expression is determined by qRT-PCR (n = 3, +/- SEM). *GAPDH* expression is used as a normalizer.

To investigate whether the *INTS8ΔEVL* mutation could have an impact on neuron differentiation we used CRISPR/Cas9-mediated genome editing to introduce a homozygous EVL deletion in mouse P19 pluripotent embryonic carcinoma cells (Fig 4A). After recombination, clonal selection and screening, we retained two lines bearing homozygous *INTS8ΔEVL* mutations (Δ*EVL*^*A*^ and Δ*EVL*^*B*^). Similarly to what we observed in fibroblast cells from patients, western blot analysis of Integrator complex subunits shows that expression levels of several subunits are reduced in the mutant cell lines compared to the parental line (Fig 4B), indicating that introduction of the ΔEVL mutation into *INTS8* is sufficient to disrupt accumulation of other members of INT. Additionally, we could also detect significant levels of U11 and U12 misprocessed snRNA in mutant cells indicating that not only is the integrity of INT disrupted in these cells but also its function (S10 Fig).

We then used these cell lines as an *in vitro* model of retinoic acid (RA)-induced neuron differentiation. After RA treatment, we followed by qRT-PCR the expression of different markers for pluripotency, retinoic acid response, neuronal precursor and neuronal differentiation. While the timing of the appearance or the morphology of differentiated neurons did not significantly differ between the two ΔEVL mutants and the parental P19 control, we observed marked differences in gene expression over the course of the process (Fig 4C). In response to RA, the induction of *hoxa1* (direct transcriptional target of RA) is markedly reduced in the mutant lines even though rapid downregulation of pluripotency genes like Oct4 is not altered. Later on, although not altered in its intensity, the induction of the neuronal stem cell marker *nestin* (*nes*) is temporally distinct between the control and the mutants, where its expression is delayed at first and later on more rapidly induced than in the control line. Finally, the induction of several neuronal markers such as *tubulin beta-3* (*tubb3*) or *reelin* is reduced while others remain similar to the control (*synapsin-1*, *syn1*). These experiments confirm that the *INTS8ΔEVL* mutant protein can have a profound effect on gene expression during a neuronal differentiation process.

## Discussion

The present study provides a rare insight into the effect of an Integrator complex deficiency in humans. We identified biallelic mutations in the Integrator complex subunit *INTS8* in three siblings and in Integrator complex subunit *INTS1* in three unrelated individuals with a rare and severe developmental brain disorder and similar phenotypic abnormalities. The *INTS1* mutation leads to strong reduction of its mRNA expression in skin fibroblasts. In the case of *INTS8*, we show that one *INTS8* mutant allele leads to rapid mRNA decay while the other translates into a protein lacking three residues in the C-terminus that impacts the overall stability of the Integrator complex. Our results indicate that Integrator-deficient patient cells contain global transcriptome perturbations manifesting as both altered splicing patterns and differential gene expression. Finally, the introduction of the *INTS8ΔEVL* mutation in P19 embryonic carcinoma cells also alters the pattern of differentiation marker expression during RA-induced neuronal differentiation.

The phenotype among the individuals with *INTS8* and *INTS1* mutations is similar, combining profound intellectual disability, epilepsy, lack of speech, facial and limb dysmorphism, altogether forming a recognizable syndrome. In patients with *INTS8* mutations, it is also associated with a rare combination of structural brain malformations including PNH and cerebellar hypoplasia. This association of PNH, a neuron migration disorder, with an Integrator subunit mutation is particularly interesting in view of the recent observation of a neuronal migration defect in mouse embryonic brain after *Ints1* and *ints11* knock-down and could implicate the Integrator complex function in the etiology of the disorder [46]. In spite of extensive search, we did not observe additional *INTS8* mutations in other individuals with similar combination of PNH and cerebellar hypoplasia, which suggests that either this association of brain malformations is not constant in this disorder or this is an ultra-rare disorder (i.e. caused by mutation in a gene with very low mutational rate) [47]. The fact that the other phenotypic features are shared with the *INTS1* mutations supports the former, while the high CADD score of the mutations and the very few *INTS8* deleterious variants reported in control populations support the latter (S2 Table). This also suggests that complete loss of *INTS8* could be incompatible with human life. The same may be true for most Integrator complex subunits as loss of any Integrator complex component tested to date has proven lethal in various animal models at early developmental stages [23–28]. Yet, it is intriguing that although *INTS8* is ubiquitously expressed, the brain is disproportionally affected by its disruption. Considering that *INTS8* expression peaks in the developing fetal brain, these observations point to a specific role for *INTS8* and more generally for the Integrator complex during brain development.

Using in-depth analysis of genome-wide alternative exon usage we show that in patient cells the splicing pattern of up to 215 individual genes is affected. This finding could be compatible with a functional defect of the spliceosome and correlates with the increased level of misprocessed UsnRNAs that we observe in patient cells. Several of these alternatively spliced genes can be individually linked to brain-related phenotypes and could therefore individually or collectively contribute to the phenotype even though no gene known to be directly involved in cortical malformation (heterotopia, with/without cerebellar hypoplasia and microcephaly) show abnormal splicing in patient fibroblasts [6]. However, among the alternatively spliced genes that we identified *SPTAN1* is of particular interest (S7 Fig). Indeed, our RNA-seq analysis indicates that exon 37 of the gene is almost completely skipped in patient cells. Patients with *SPTAN1* mutations present severe intellectual disability, no visual tracking, epilepsy and spastic tetraplegia. Brain imaging shows cerebellar hypoplasia, acquired microcephaly and hypomyelination [48]. Hence, patients with *SPTAN1* mutations share many rare features with the patients described in this study. Also *RPGRIP1L*, mutated in Joubert syndrome with cerebellar hypoplasia, retinal dystrophy and variable cortical malformation, has disrupted alternative splicing in patients with *INTS8* mutations. Although we find a broad disturbance of splicing patterns, it is only speculative to relate the results of individual genes in fibroblast cell lines to brain development. Numerous studies have however demonstrated that the brain relies heavily on alternative splicing to regulate neuronal development [49–54]. Moreover, spatiotemporal control of alternative splicing is crucial in the generation and differentiation of neuronal progenitors [55]. This is supported by the finding that mutations in the splicing factor RNA-binding motif *RBM10* cause abnormal mRNA splicing, microcephaly, PNH, and cerebellar hypoplasia [56]. In addition, alterations in UsnRNAs have severe consequences on brain development in both mice and humans. A mutation in the mouse *Rnu2-8* gene, coding for U2 snRNA, results in abnormal pre-mRNA splicing of specific transcripts and in cerebellar degeneration [29]. Similarly, mutations in the minor spliceosome *U4atac* snRNA gene in humans result in splicing defects and the rare disorder microcephalic osteodysplastic primordial dwarfism type I (MOPD1) [30, 57]. Mutations in another minor spliceosome snRNA, *RNU12*, result in U12-type exon retention and are associated with cerebellar ataxia[31]. Therefore, a toxic effect of the accumulation of misprocessed UsnRNAs on the spliceosome in response to *INTS8* mutations is possible and could result in the differential splicing patterns that we observed in patient versus control cells.

Our results show a great number of differentially expressed genes in the patient cells. Significantly and reproducibly the top dysregulated genes in exon arrays are *NOG* (OMIM 602991, encoding noggin) and *TUBA1B* that both have a primary role in brain and development (S7 Fig). Noggin inhibits BMP4, one of the major bone morphogenic proteins required for growth and patterning of neural tube while *TUBA1B* (OMIM 602530, coding for tubulin-beta) shows its highest expression in brain and is one of the several tubulin genes essential for normal cortical development in human. Tubulin-beta forms a dimer with tubulin-alpha, encoded by *TUBA1A*, a gene mutated in syndromic cortical malformation with microcephaly, cerebral and cerebellar dysgyria [7]. On a mechanistic level, the effects of *INTS8* mutation on gene expression could be linked to the newly identified function of Integrator complex in the regulation of RNAPII-dependent transcriptional initiation, pause-release and elongation of protein-coding genes [14, 16, 17]. This finding greatly expanded the scope of Integrator complex function as well as its potential impact on transcription, particularly on genes known to be regulated by promoter-proximal pausing such as immediate early genes (IEGs) [14]. Moreover, at the level of the neuron itself, promoter-proximal pausing and IEGs play an important role in neuronal development, synapse plasticity and maturation through neuronal activity-dependent transcription activation [39–41]. It is therefore possible that part of the disease pathogenesis associated with *INTS8* mutations also results from widespread transcription deregulation, apart from abnormal splicing.

A definitive answer will require a detailed analysis of *INTS8* role in neuronal differentiation and in brain development. Our results in P19 differentiation by retinoic acid indicates that *INTS8ΔEVL* mutation can indeed cause misprocessing of snRNA and can affect expression of many differentiation-regulated genes and could therefore have consequences on neuronal and brain development. The recent progress in genetic engineering ushered by the development of CRISPR/Cas9-based genome editing tools will enable the development of animal models tailored in the future to address these questions. Likewise, progress in cell reprogramming and induced pluripotent cell production should allow for the direct use of patient cells to study the impact of *INTS8* mutations on neuron differentiation in their original genetic background.

Our study provides the first evidence for a crucial role of the Integrator complex during human brain development. *INTS8* is essential for the structural and functional integrity of Integrator complex, and mutated *INTS8* causes increased UsnRNA misprocessing, increased AS events and altered gene expression, confirming a central role for Integrator complex in transcriptional regulation and, together with *INTS1* mutations, an unexpected role in human brain development.

## Materials and methods

### Ethics statement

All study participants or their legal caretakers gave written informed consent to participate in this study, and for publication of images, according to Erasmus MC institutional review board requirements (protocol METC-2012387).

### Genomic analysis: Whole genome sequencing, whole exome and Sanger sequencing

Details of the Whole-genome sequencing (WGS), whole-exome sequencing (WES) and Sanger sequencing are provided in Supplemental methods (S1 Text). Data are deposited internally at the Erasmus MC in respect to the privacy of the families.

### qRT-PCR of endogenous INTS8

Quantitative qRT-PCR of RNA extracted from cultured fibroblasts of the affected siblings, their unaffected brother, and two control cell lines was carried out using a KAPA SYBR FAST qPCR Kit (Kapa Biosystems) in the CFX96 Real-Time system (BioRad). Details are provided in S1 Text.

### qRT-PCR of misprocessed UsnRNA and Integrator target gene expression in primary fibroblasts

qRT-PCR was performed using RNA from cultured fibroblasts on a Stratagene Mx3000P real-time PCR system (Agilent) using the KAPA SYBR FAST qPCR Kit (Kapa Biosystems) according to manufacturer’s instructions. Details are provided in S1 Text.

### Splicing assays

Exon 8 and flanking intronic sequences of human *INTS8* gene were amplified from HEK293T genomic DNA by PCR (primers in S1 Text). The amplicon was cloned into the pGint vector [58] using BamHI and SalI restriction sites. The A893G mutation was introduced by site directed mutagenesis (primers in S1 Text). The resulting constructs were transfected in HEK293T and HeLa cells using Lipofectamine2000 (Thermo Fisher). After 48h, total RNA was extracted, purified and used to generate cDNA using M-MLV reverse transcriptase (Thermo Fisher) using manufacturer specifications. PCR amplification of the corresponding splicing product was performed. For detection and quantification, oligonucleotides were 5’ radiolabeled using 32P-γATP and T4 Polynucleotide Kinase (Thermo Fisher) and added in a 1/10 ratio with unlabeled oligonucleotides. The corresponding PCR reactions were resolved onto a 6% non-denaturing acrylamide gel, fixed and dried. The gels were scanned using a storage phosphor screen and a Storm scanner (GE Healthcare) and quantified using ImageQuant software (GE Healthcare).

### INTS8 stable cell lines and Flag-affinity purification

The human *INTS8* cDNA was amplified by PCR from HeLa cell cDNA and the corresponding PCR product was cloned into a modified pCDNA6 plasmid containing an N-terminal 3XFlag tag (See S1 Text). The EVL deletion was introduced by site-directed mutagenesis. HEK293T cells were transfected with either construct. Flag-affinity purification was performed as in [11] using nuclear extracts from approximately 10^9^ cells. Integrator subunits in the eluate were detected by Western blot using the following antibodies: anti-FLAG M2 (Sigma), INTS1 (Bethyl, A300-361A), INTS3 (Bethyl, A302-050A), INTS4 (Bethyl, A301-296A), INTS5 (Abcam, ab74405), INTS9 (Bethyl, A300-422A), INTS11 (Bethyl, A301-274A) and INTS12 (Proteintech, 16455-1-AP). Oligonucleotide sequences are listed in S1 Text.

### Western blot of Integrator complex components

Cultured fibroblasts were used for western blots of Integrator complex components. Approximately 32 μg of the clarified whole cell extract were separated on an 8% acrylamide SDS-PAGE gel. After transfer to a PVDF membrane, the presence of the different Integrator complex subunits was assessed by Western blot using the antibodies listed above.

### Exon arrays

Six Affymetrix GeneChip Human Exon 1.0 arrays of RNA extracted from cultured fibroblasts of all three affected siblings and three age and sex-matched controls were robust multi-array (rma) normalized on transcript level using the R package (http://www.r-project.org). Top down- and upregulated genes with p values <0.02 were analyzed for enriched gene ontology terms (goterms_BP_all) using DAVID (medium stringency) to identify clusters with significant enrichment (enriched score ≥ 1.3). CEL file microarray data are available under GEO accession number GSE48849 (http://www.ncbi.nlm.nih.gov/geo/).

### RNA-Seq analysis

Poly(A) mRNA fractions isolated from cultured fibroblasts from patient III-2, III-4, and two controls were subjected to RNA-Seq analysis. In order to detect even slight changes in splicing, we utilized high depth RNA sequencing (S4 Table). The resulting data was analysed using a recently developed pipeline specifically designed to monitor splicing efficiency [59]. In addition, we analysed patient and control RNA-Seq data for differences in gene expression at steady state levels using EdgeR. Data are deposited in Gene Expression Omnibus (GSE76878).

### P19 RA-induced differentiation assay

Differentiation of P19 cells was conducted according to [60]. Briefly, 10^7^ exponentially growing cells were seeded on agarose-coated plate in a culture medium without serum containing 1μM all-trans Retinoic Acid (sigma) and N-2 supplement (Thermo Fischer). After 48h, embryonic bodies were collected and seeded on tissue culture plates in medium with serum. After 48h, medium was replaced with culture medium without serum containing N-2 supplement. Half of the culture medium was replaced every 48H with fresh medium. RNA was extracted at day0, day2, day4 and day8 using Trizol (Thermo Fisher). The mRNA expression levels of neuronal differentiation markers were monitored by qRT-PCR (see S1 Text for details).

### CRISPR/Cas9-mediated genome editing in P19 cells

Genome editing was conducted as in [61] with minor variations. See S1 Text for details.

## Supporting information

S1 Text Supplemental methods. (DOCX)

S1 Fig qRT-PCR of INTS1 expression in cultured fibroblasts. (PDF)

S2 Fig INTS8 variants 3 transcript is subject to NMD. (PDF)

S3 Fig WT and mutant INTS8-associated peptides. (PDF)

S4 Fig Expression of mature U snRNA in patient and control fibroblasts. (PDF)

S5 Fig qRT-PCR for endogenous *ILR7*, *TUBA1B* and *NOG* expression. (PDF)

S6 Fig qRT-PCR validation of differential gene expression in patient fibroblasts, selected from RNASeq. (PDF)

S7 Fig Analysis of splicing alterations in patient cells. (PDF)

S8 Fig Expression of *INTS8* during fetal development. (PDF)

S9 Fig RNA sequencing of human brain tissues ate several developmental stages. (PDF)

S10 Fig qRT-PCR showing misprocessed U11 and U12 in RNA extracts of WT and mutant P19 cells. (PDF)

S1 Table Data regarding whole-genome sequencing including coverage, number of variants per individual. (DOCX)

S2 Table In silico prediction results of the two INTS8 mutations. (XLSX)

S3 Table List of genes with significantly different gene expression in patient cultured fibroblasts vs controls, results from the exon arrays (top 682 genes show p< 0.02). (DOCX)

S4 Table RNAseq alignment statistics. (XLSX)

S5 Table List of genes with significantly different gene expression in patient cultured fibroblasts vs controls in the RNAseq (top 3002 genes show p<0.02). (DOCX)

S6 Table List of overlapping genes with significantly different gene expression in patient cultured fibroblasts vs controls in both the exon arrays and the RNAseq (obtained after cut-off point of p<0.05 and ranked on Sum.logFC). (XLS)

S7 Table Alternative exon usage in patient cultured fibroblasts vs controls, results from RNAseq. (XLS)

S8 Table WGS–list of variants. (XLS)
