# Correction: Human mutations in integrator complex subunits link transcriptome integrity to brain development

**DOI:** 10.1371/journal.pgen.1006923

**Published:** 2017-08-01

**Authors:** Renske Oegema, David Baillat, Rachel Schot, Leontine M. van Unen, Alice Brooks, Sima Kheradmand Kia, A. Jeannette M. Hoogeboom, Zheng Xia, Wei Li, Matteo Cesaroni, Maarten H. Lequin, Marjon van Slegtenhorst, William B. Dobyns, Irenaeus F. M. de Coo, Debbie van den Berg, Frans W. Verheijen, Andreas Kremer, Peter J. van der Spek, Daphne Heijsman, Eric J. Wagner, Maarten Fornerod, Grazia M. S. Mancini

Dr. Debbie van den Berg should be included in the author byline instead of the Acknowledgments. She should be listed as the fifteenth author, and her affiliation is: Neural Stem Cell Biology Laboratory, The Francis Crick Institute, London, United Kingdom. Her current address should be: Department of Cell Biology, Erasmus Medical Center, Rotterdam, The Netherlands. The contribution of this author is as follows: Investigation.
