# Supplementary material for: Human mutations in integrator complex subunits link transcriptome integrity to brain development
Source: PLoS Genet. 2017 May 25;13(5):e1006809. doi: 10.1371/journal.pgen.1006809 (PMC5466333; doi:10.1371/journal.pgen.1006809)
Supplement: S1 Text — (DOCX) [file pgen.1006809.s001.docx]

**Oegema et al.**

**Supplemental Methods**

**WES analysis**

Genomic DNA was isolated from peripheral blood leukocytes of family trios (proband and both parents) and exome-coding DNA was captured with the Agilent Sure Select Clinical Research Exome (CRE) kit. Reads were aligned to Hg19 using BWA (BWA-MEM v0.7.5a) and variants were called using the GATK haplotype caller (v2.7-2). Detected variants were annotated, filtered and prioritized using the Bench lab NGS v.3.1.2 platform (Cartagenia, Leuven, Belgium).

**WGS analysis**

Analysis of the WGS data for the four sibling and their parents was done using cgatools (http://www.completegenomics.com/sequence-date/cgatoools) and TIBCO / Spotfire (http://spotfire.tibco.com). Data of all family members was compared and tested for 3 inheritance models: compound heterozygous, homozygous and de novo heterozygous. Only non-synonymous variants, variants disrupting splice sites and small insertions and deletions (up to approximately 50 bp) were included. Reference data from international (dbSNP, ExAC) and in-house databases* were used to exclude common variants and non-pathogenic variants. *De novo* analysis did not result in any variants. For the other variants present after these filtering steps (Table S8), the raw data were manually checked for segregation and frequency data. From these, *INTS8* was the only plausible candidate gene following the compound heterozygous inheritance model.

* Stubbs A, McClellan E, Horsman S, Hiltemann S, Palli I, Nouwens S, Koning A, Hoogland F,

Reumers J, Heijsman D, Swagemakers S, Kremer A, Meijerink J, Lambrechts D, van der Spek P.

Huvariome: a web server resource of whole genome next-generation sequencing allelic

frequencies to aid in pathological candidate gene selection. J Clin Bioinformatics 2:2-19 2012.

**Sanger sequencing**

**A**mplification reactions were performed in a total volume of 20 μl, containing 1× PCR buffer with Mg (Roche), 200 μM of each dNTP, 1 μM forward primer, 1 μM reverse primer, 0.1 units Fast Start Taq DNA polymerase (Roche), and 25 ng genomic DNA. PCR conditions were as follows: 5′ 96°C, 10 cycles of 30″ 96°C, 30″ 68°C (−1°C/cycle), 60″ 72°C, followed by 25 cycles of 30″ 96°C, 30″ 58°C, 60″ 72°C, and a final extension for 5′ 72°C.

PCR reactions were purified with ExoSAP-IT (USB). Direct sequencing of both strands was performed with Big Dye Terminator chemistry (version 3.1; Applied Biosystems). DNA fragment analysis was performed with capillary electrophoresis on an ABI 3130 Genetic Analyzer (Applied Biosystems) with the software package Seqscape (Applied Biosystems, version 2.1).

Primers used for confirmation of mutations with Sanger:

INTS8_exon8F AGCCCGGCTTGAATTTTTCG

INTS8_exon8R AAATGACTTGCCCCAAAACG

INTS8_exon27F TTCCAAGACTCCTGGGTTTTACAG

INTS8_exon27R CAAGACTCCTGGGTTTTACAGTTAAGAGG

**qRT-PCR for endogenous *INTS8* expression**

RT-qPCR was carried out using a KAPA SYBR FAST qPCR Kit (Kapa Biosystems) in the CFX96 Real-Time system (BioRad). Thermal cycling conditions were as follows: a denaturing step (95°C for 3 min), followed by 35 cycles of denaturing (95°C for 5”), annealing and extension (60°C for 30”). Fluorescence detection and data analysis were performed by BioRad CFX Manager 2.0. Experiments were performed in triplicate with the use of UBE2D2 (Entrez GeneID 7322 [MIM 602962])and GAPDH (Entrez GeneID 2597 [MIM 138400]) as a reference for gene expression normalization.

INTS8_qrt_1F GAACACATTTCTATTTGGCCATCTT

INTS8_qrt_1R TCGAACTAGCTCTTTCACTGTGATTG

INTS8_qrt_2F AAAGAGGAGAAACAGATAAAAGACAAATTG

INTS8_qrt_2R GCTGTAACACTTCTTCTGGATTGC

UBE2D2_F GATCACAGTGGTCTCCAGCA

UBE2D2_R CGAGCAATCTCAGGCACTAA

GAPDH_F GGAAGAGAGAGACCCTCACTGC

GAPDH_R CCCTCAGATGCCTGCTTCA

The same method was used for qRT-PCR of endogenous *INTS1* expression in patient fibroblasts (primer sequences available upon request), with the only difference that *CLK2* was used as reference gene.

**Western blot of INTS8 in control and in patient fibroblasts:**

Methods: A cell pellet of fibroblasts (approx. 3.10^6^  cells) were lysed by the nuclear extraction protocol of Life technologies. Samples were run on a 4-12% gradient gel, 32µg of total protein/lane. INTS8 was detected in the nuclear fraction by the rabbit polyclonal LL20530 (ab8728, Abcam) and actin by mouse monoclonal α-actin, clone A40 (Sigma). Specificity of the INTS8 antibody was confirmed by performing a knockdown on control fibroblasts using SMARTpool siGENOME Human INTS8 siRNA (Dharmacon, M-020270-01-0010) (data not shown).

**Immunocytochemistry**

Rabbit polyclonal antibodies anti human INTS8 (ab 87282, Abcam, 1/200 dilution), anti human INTS11 (ab84719, Abcam, 1/200 dilution), and mouse monoclonal antibodies against SMN (anti-Gemin 1 antibody [2B1]; ab5831, Abcam, 1/100 dilution) were used. The Hoechst reagent was used to stain nuclei. SMN was used as marker of nuclear foci of active post-transcriptional RNA processing, such as the gems and Cajal bodies. Near confluent fibroblast cultures were fixated with 4% PFA and incubated in blocking buffer (50 mM Tris pH7.4, 0.9% NaCl, 0,25% gelatine, 0,5% TritonX100) at 4°C overnight. Fluorescence was visualized on a Zeiss Imager Z1 with a AxioCam MRM/ ICc1 (Zeiss).

We cloned INTS8 cDNA that was either wild-type (WT) or lacking the EVL amino acids (ΔEVL) into a vector that expressed the full-length protein fused to an N-terminal 3XFLAG tag. These constructs were transfected into HEK293T cells and a stable population of cells was selected using blasticidin resistance. These stable lines were then expanded, used to produce nuclear extracts, and finally INTS8-associated proteins were isolated by affinity purification using anti-FLAG M2 agarose beads. Purified complexes were separated using SDS-PAGE followed by silver staining and a very similar pattern of associated peptides was observed.

**RT-PCR quantification of misprocessed UsnRNA and Integrator target gene expression in primary fibroblasts.**

Misprocessing of U snRNA and gene expression were assessed with qRT-PCR using RNA extracted from cultured primary fibroblasts using Trizol (Thermo Fischer). After DNAseI (Thermo Fischer) treatment, cDNA was synthetized from 1 μg of total RNA using random hexamers and M-MLV reverse transcriptase (Thermo Fischer) according to the manufacturer’s instructions. Levels of expression were quantified on a Stratagene Mx3000P real-time PCR system (Agilent) using the KAPA SYBR FAST qPCR Kit (Kapa Biosystems) according to manufacturer’s instructions. 7SK (UsnRNA and gene expression) and UBE2D2 (gene expression only) were used as references for normalization.

Primers for snRNA missprocessing qRT-PCR:

U1 (5’- AATGTGGGAAACTCGACTGC-3’, 5’- TGCAGGCGACATGTTACTTC-3’),

U2 (5’- CTTCGGGGAGAGAACAACC-3’, 5’- GACACTCAAACACGCGTCA-3’),

U4 (5’- GCATTGGCAATTTTTGACAG-3’, 5’- GAACCCCGGACATTCAATC-3’),

7SK (5’- AGGACCGGTCTTCGGTCAA-3’, 5’- TCATTTGGATGTGTCTGCAGTCT-3’).

Primers for gene expression qRT-PCR:

CLK1 (5’- CCCGTGATTGCGTTACAAGC-3’, 5’- CCTGGGCACTGCTATGTGA-3’),

DHRS3 (5’- GGAGAACGTCCTCATCACCG-3’, 5’- CGTCGTCTCCTTCAGGCATT-3’),

DSP (5’- CAACAACCGGACCCTGGAAC-3’, 5’- ACCACCTGAGTACACTGATTCTTT-3’),

GAPDH (5’- GGAAGAGAGAGACCCTCACTGC-3’, 5’- CCCTCAGATGCCTGCTTCA-3’),

NEDD9 (5’- CCGCAGTGCTTAATGCTGTC-3’, 5’- CAGTTCCTCGGCACACTCTG-3’),

NPTX1 (5’- ACCGAGGAGAGGGTCAAGAT-3’, 5’- CAGGGCGGTTGTCTTTCTGA-3’),

NTN1 (5’- CCTCCAAGGGGAAGCTGAAG-3’, 5’- CTTGTCCGCCTTCAGGATGT-3’),

OSR2 (5’- GCTCTCGCTTTAGCTGCTCG-3’, 5’- CCCAGTGAGACAACAGCACG-3’),

PLOD2 (5’- CTGGAATCCCTGTCTGGGTG-3’, 5’- TGACCAAGGACCTTCACAGT-3’),

PPM1L (5’- TCCCAGAGGCCCTTAAACAG-3’, 5’- GTTGGCCACAGTGAGGTCTT-3’),

PRSS23 (5’- CCACACCTGTCTGAGCGG-3’, 5’- AGGAAGAGGAGCCCTGGAAT-3’),

RDH10 (5’- AGGCTGCTGAAAAGGATGGAA-3’, 5’- GAGGTGGCAGAAAAGGCTCA-3’),

SEPRINE1 (5’- AGCCACTGGAAAGGCAACAT-3’, 5’- GTCGACTTCAGTCTCCAGGG-3’),

SLC38A1 (5’- CGAGGGTAAAAACCCCGGAA-3’, 5’- AGCTTGACACCCCTGTTAGC-3’),

SGK1 (5’- GGCATGGTGGCAATTCTCAT-3’, 5’- TCAGGCTCCTGAGGTTGGG-3’),

SOX4 (5’- ACCTGAACCCCAGCTCAAAC-3’, 5’- GTGCAGTAGTCCGGGAACTC-3’),

TNFRSF21 (5’- ATTGTGGAAAAGGCAGGGCT-3’, 5’- CTTTCCACTGGCTTCCCACT-3’),

UBE2D2 (5’- GATCACAGTGGTCTCCAGCA-3’, 5’- CGAGCAATCTCAGGCACTAA-3’).

**RT-PCR quantification of neuronal differentiation marker mRNA expression in P19 cells.**

Total RNA was extracted using the Trizol (Thermo Fisher) according to the manufacturer’s instructions. After DNAseI (Thermo Fischer) treatment, cDNA was synthetized from 1 μg of total RNA using random hexamers and M-MLV reverse transcriptase (Thermo Fischer) according to the manufacturer’s instructions. Levels of expression were quantified on a CFX Connect Real-Time PCR Detection System (Biorad) using the iTaq™ universal SYBR® Green supermix (Biorad) according to manufacturer’s instructions. GAPDH were used as reference for normalization.

Reelin (5’-TGGACCATGTGGAGGTCG-3’, 5’-TGGATTCTTCATGGGTATCGCC-3’)

Syn1 (5’-GTCTACCTTGACCTTGCCCA-3’, 5’-CAAGAAGCTTGGAACAGAGGA-3’)

Gapdh (PrimePCR Assay Gapdh, Mmu, qMmuCED0027497, Biorad)

Oct4 (PrimePCR Assay Pou5f1, Mmu, qMmuCED0025033, Biorad)

Hoxa1 (PrimePCR Assay Hoxa1, Mmu, qMmuCED0005091, Biorad)

Nes (PrimePCR Assay Nes, Mmu, qMmuCID0023067, Biorad)

Tubb3 (PrimePCR Assay Tubb3, Mmu, qMmuCID0018119, Biorad)

**Northern blot analysis**

One microgram of total RNA was resolved on a 6% acrylamide/7M urea denaturing gel and transferred to a Hybond-N+ membrane (GE healthcare) at 80V for an hour in 0.5X TBE. The transferred RNA was UV crosslinked twice with 120mJ/cm2 at 254nm. The membrane was blocked for 30min at 50^o^ C in 5 ml Quickhyb buffer (Agilent) after which 2.10*^6^ cpm of the corresponding radiolabeled probe were added and let to hybridize for 16 hour at 50^o^ C. After hybridization, the membrane was washed in 1X SSC with 0.1% SDS for at least an hour at 50^o^ C. Radioactivity was detected by autoradiography and quantified using a Storm Phosphorimager system (GE Healthcare). U6 was used as a reference for quantification.

Radiolabeled probes: DNA templates were amplified by PCR using the following primer pairs:

U1 (5’-CACAAATTATGCAGTCGAGTTTCC-3’, 5’-TTTGGCTAAGATCAAGTGTAGTATCTGTTC-3’),

U2 (5’-AATCCATTTAATATATTGTCCTCGGATAGA-3’, 5’- AATCCATTTAATATATTGTCCTCGGATAGA -3’),

U4 (5’- GCGCGATTATTGCTAATTGAAA-3’, 5’- AAAAATTGCCAATGCCGACTA-3’),

U6 (5’- GCTTCGGCAGCACATATACTAAAAT-3’, 5’- ACGAATTTGCGTGTCATCCTT-3’),

U11 (5’- GTGCGGAATCGACATCAAGAG-3’, 5’- CGCCGGGACCAACGAT-3’),

U12 (5’- AACTTATGAGTAAGGAAAATAACGATTCG-3’, 5’- CGACCTTTACCCGCTCAAAA-3’). Radiolabeled probes were generated using the Prime-it II Random Primer Labeling kit (Agilent) according to the manufacturer’s instructions.

**Splicing assays**

Exon 8 and flanking intronic sequences of human *INTS8* gene were amplified from HEK293T genomic DNA by PCR using the following primers: 5’-gactggatccgtgatccgccagtctcggcctc-3’ and 5’-gatcgtcgacgaacttagttttgtctggcttcc-3’. The corresponding amplicon was cloned into the pGint vector ([Bonano et al., 2007](#_ENREF_3)) using BamHI and SalI restriction sites. The A893G mutation was introduced by site directed mutagenesis using the following oligonucleotides: 5’-CATTATCTCTTCATTGTACCATAGGTGAGAAGCGGTTAGCTGGC-3’ and 5’-GCCAGCTAACCGCTTCTCACCTATGGTACAATGAAGAGATAATG-3’.

The resulting constructs were transfected in HEK293T and HeLa cells using Lipofectamine2000 (Thermo Fisher). Empty pGint was used as a control. After 48h, total RNA was extracted using Trizol (Ambion), treated with DNAse I and purified by phenol/chloroform extraction. Two micrograms of RNA were used to generate cDNA using M-MLV reverse transcriptase (Thermo Fisher) and random hexamers according to manufacturer’s instructions. PCR amplification of the corresponding splicing product was performed using the following oligonucleotides: GFP_F (ACGTAAACGGCCACAAGTTC) and GFP_R (TGAACTTCAGGGTCAGCTTG). For detection and quantification, oligonucleotides were 5’ radiolabeled using 32P-γATP and T4 Polynucleotide Kinase (Thermo Fisher) and added in a 1 to 10 ratio with unlabeled oligonucleotides. The corresponding PCR reactions were resolved onto a 6% non-denaturing acrylamide gel, fixed and dried. The gels were scanned using a storage phosphor screen and a Storm scanner (GE Healthcare) and quantified using ImageQuant software (GE Healthcare). pGint was a gift from Mariano Garcia-Blanco (Addgene plasmid # 24217).

Endogenous splicing analysis was performed as above using the following primers:

INTS8_F:GAAATGCAGTGCCAGGTGTG

INTS8_R: GACAATAGCCAGCTAACCGC

ADAM15_F: TCCTCAGCCTCCTGGTCTTA

ADAM15_R:GGCAGGCAGTGGCTTCCTTG
ATL3_F: GACTGTGCTACCATCTTTGCTC

ATL3_R:TGAATGTGATTTCGAACATTCTG

FBLN2_F: GCTCAGCCATATGCTCCTGT

FBLN2_R: TGAGTGCCTTGTAGCAGTGG

TMEM119_F: GCACGGACTCTCTCTTCCAG

TMEM119_R: GCAGCAACAGAAGGATGAGG

MTHSFD_F:ACAAATTTGGGGCTACATGG

MTHSFD_R: AACAATCCCGTTCTCAGTCG.

Where indicated, cells were treated 6 hours before extraction by addition of 100 ug/mL puromycin (Sigma) or 30 ug/mL cycloheximide (Sigma) to the culture media in order to block translation and nonsense-mediated decay.

**Stable cell lines and Flag-affinity purification**

The human INTS8 cDNA was amplified by PCR from HeLa cell cDNA using the following oligonucleotides: 5’-GATCGAATTCTGAGCGCGGAGGCGGCGGAC-3’ and 5’-GATCCTCGAGTTAAAAGTAAAGTTTTGCCATTGC-3’. The corresponding PCR product was cloned into a modified pCDNA6 plasmid containing an N-terminal 3XFlag tag using EcoRI and XhoI restriction sites. The EVL deletion was introduced by site directed mutagenesis using the following oligonucleotides: 5’-gttgaatgcaagcaatccagaacagctggcagcgcagagaagg-3’ and 5’-ccttctctgcgctgccagctgttctggattgcttgcattcaac-3’. HEK293T cells were transfected with either construct. Forty eight hours after transfection, cells were selected with 10 ug/mL Blasticidin S (Thermo Fisher). Single clones were isolated by limit dilution and screened for Flag-INTS8 expression by Western blot.

Flag-affinity purification was performed as in ([Baillat et al., 2005](#_ENREF_2)) using nuclear extracts from approximately 10^9^ cells. Integrator subunits in the eluate were detected by Western blot using the following antibodies: anti-FLAG M2 (Sigma), INTS1 (Bethyl, A300-361A), INTS3 (Bethyl, A302-050A), INTS4 (Bethyl, A301-296A), INTS5 (Abcam, ab74405), INTS9 (Bethyl, A300-422A), INTS11 (Bethyl, A301-274A) and INTS12 (Proteintech, 16455-1-AP).

**CRISPR/Cas9-mediated genome editing in P19 cells.**

P19 cells were maintained in DMEM medium supplemented with 10% fetal bovine serum, 2 mM glutamine, 2mM sodium pyruvate and antibiotic/antimycotic (Thermo Fisher Scientific). Cells were plated into a 24-well dish the day prior to transfection. The following day, the cells were transfected using Lipofectamine 2000 (Thermo Fisher Scientific) with 250 ng pST1374-NLS-FLAG-linker-Cas9, 250 ng of the pGL3-U6-sgRNA-PGK-puromycin containing the sgRNA sequence for *Ints8* and 10 pmol of the ssODN as an HR donor:

sgRNA^A^: 5’-CCCTGAAGAAGTATTACAGC-3’

sgRNA^B^: 5’-CCAGCTGTAATACTTCTTCA-3’

ssODN donor : TTATGTGTACTACTGTGACAATTTTTTTCAGTCAAACTTCTAATAACACTTTTCTGTTTTAAGATCAAAGCCATTGGCCAAACAGAATTGAATGCTAGCAACCCTGAACAGCTGGCAGCACAGAGAAGGAAAAAGAAGTTTCTACAAGCAATGGCAAAACTTTACTTTTAAGCAGTTAGTTAATT

After 24 h, the cells were expanded into a 6 well dish. Antibiotic selection using 10 mg/mL blasticidin (Thermo Fisher Scientific) and 2.5 mg/mL puromycin (Sigma Aldrich) was initiated 48 h after transfection and prolonged for 48 h. After 48 h of selection, transfected cells were maintained in DMEM medium supplemented with 10% fetal bovine serum and antibiotic/antimycotic. Clonal cell lines were obtained by limiting dilution in 96 well dishes at an initial dilution ration of 0.5 cell per well. After clonal expansion Genomic DNA was isolated from approximately 10^6^ cells using the RGDE protocol (SM Ali et al., Forensic Science International: Genetics supplement series (2008) 63-65). Ints8ΔEVL deletion was detected by PCR followed by NheI digestion using the following oligonucleotides:

5’-TGTCCCTCCAGGTAACCATACT-3’ and 5’-AATTTGTTTCAGTTTGTTTTGGTCT-3’.

For sequencing purposes, PCR amplicons were cloned using the Zero Blunt TOPO PCR Cloning Kit (Thermo Fischer). Positive clones were submitted to Sanger sequencing using the M13R primer (data not shown).

pST1374-NLS-flag-linker-Cas9 was a gift from Xingxu Huang (Addgene plasmid # 44758). pGL3-U6-sgRNA-PGK-puromycin was a gift from Xingxu Huang (Addgene plasmid # 51133).
