# Supplementary material for: Human mutations in integrator complex subunits link transcriptome integrity to brain development
Source: PLoS Genet. 2017 May 25;13(5):e1006809. doi: 10.1371/journal.pgen.1006809 (PMC5466333; doi:10.1371/journal.pgen.1006809)
Supplement: S1 Fig — (PDF) [file pgen.1006809.s002.pdf]

Figure S1.

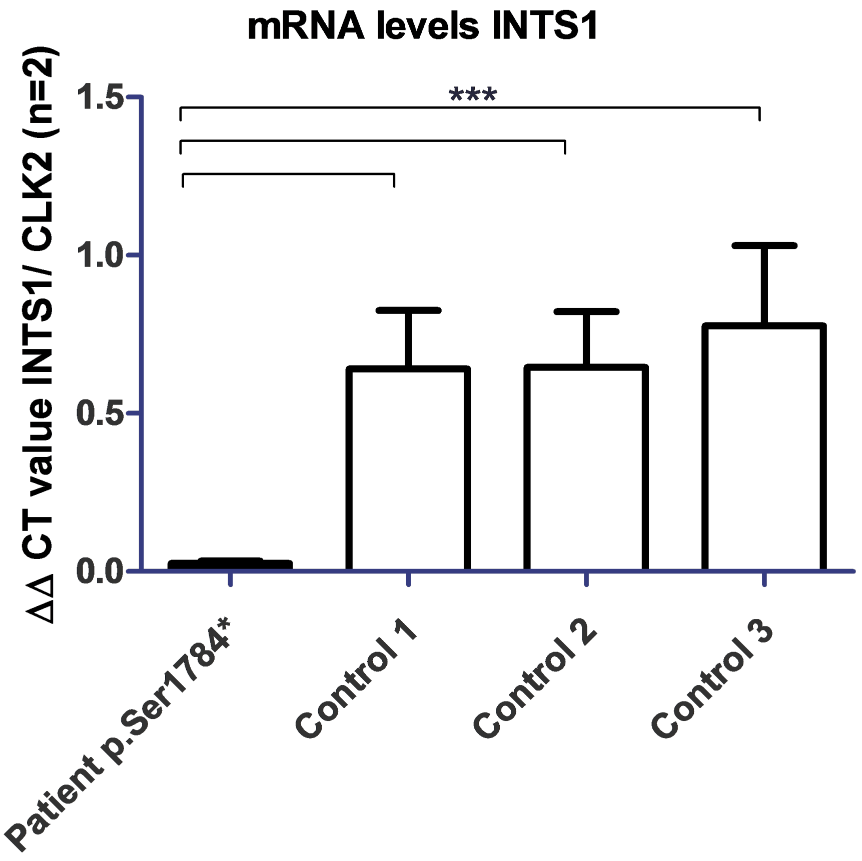

Legend for Fig. S1: qRT-PCR of *INTS1* expression in cultured skin fibroblasts from patient 2 with homozygote p.Ser1784* mutation. The expression level is normalized to *CLK2* expression.
