# Supplementary material for: Human mutations in integrator complex subunits link transcriptome integrity to brain development
Source: PLoS Genet. 2017 May 25;13(5):e1006809. doi: 10.1371/journal.pgen.1006809 (PMC5466333; doi:10.1371/journal.pgen.1006809)
Supplement: S2 Fig — (PDF) [file pgen.1006809.s003.pdf]

Figure S2

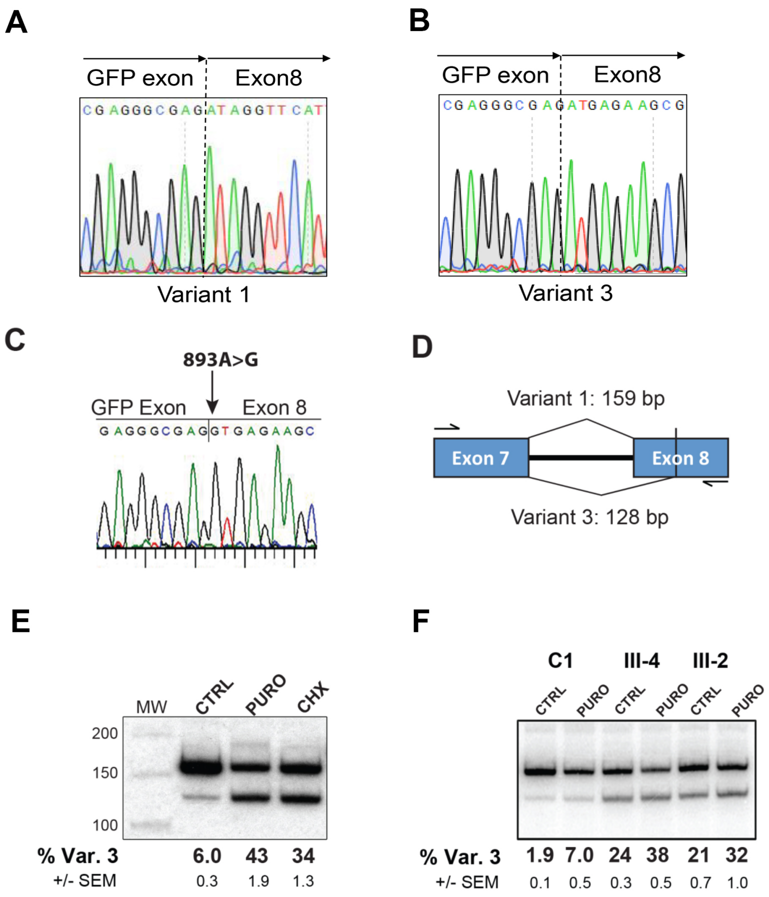

Legend for Fig. S2. INTS8 variant 3 transcript is subject to NMD.

A, B and C. Sanger sequencing. Amplicons from the splicing assay using the reporter constructs described in Fig. 2C were cloned and sequenced to verify the exon-exon junction. A: variant 1 transcript from the INTS8 exon8 wild-type construct. B: variant 3 transcript from the INTS8 exon8 wild-type construct. C: transcript from the INTS8 893A>G exon8 construct. D. Schematic of *INTS8* genomic organization showing the position of variant 1 and variant 3 splice sites. PCR primers have been designed in exon7 and exon8 to detect alternative splicing. The size (in bp) of the amplicons corresponding to each variant is indicated. E. HeLa cells were treated with 100 µg/mL puromycin or 30 µg/mL cycloheximide for 6 hours. Total RNA was extracted, reverse transcribed and analysed by PCR using ^{32}P radiolabeled primers to detect INTS8 exon8 splicing pattern. After non-denaturing PAGE and quantification, variant 3 to variant 1 ratio was calculated (n=3). F. Patient and control cells were treated with 100 µg/mL puromycin for 6 hours. Total RNA was extracted, reverse transcribed and analysed by PCR using ^{32}P radiolabeled primers to detect INTS8 exon8 splicing pattern. After non-denaturing PAGE and quantification, variant 3 to variant 1 ratio was calculated (n=3).
