# Supplementary material for: Human mutations in integrator complex subunits link transcriptome integrity to brain development
Source: PLoS Genet. 2017 May 25;13(5):e1006809. doi: 10.1371/journal.pgen.1006809 (PMC5466333; doi:10.1371/journal.pgen.1006809)
Supplement: S3 Fig — (PDF) [file pgen.1006809.s004.pdf]

Figure S3.

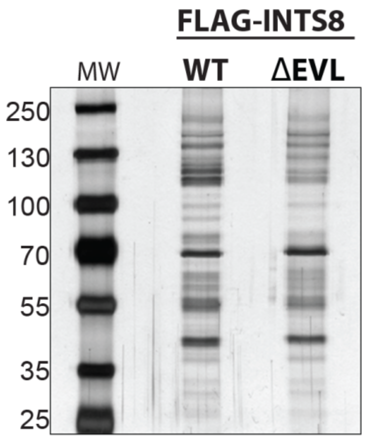

Legend for Fig. S3. WT and mutant INTS8-associated peptides.

Eluates from FLAG-INTS8 affinity purification of nuclear extracts from stably expressing HEK293T cell lines were resolved by SDS-PAGE and silver stained.
