# Supplementary material for: Human mutations in integrator complex subunits link transcriptome integrity to brain development
Source: PLoS Genet. 2017 May 25;13(5):e1006809. doi: 10.1371/journal.pgen.1006809 (PMC5466333; doi:10.1371/journal.pgen.1006809)
Supplement: S4 Fig — (PDF) [file pgen.1006809.s005.pdf]

Figure S4.

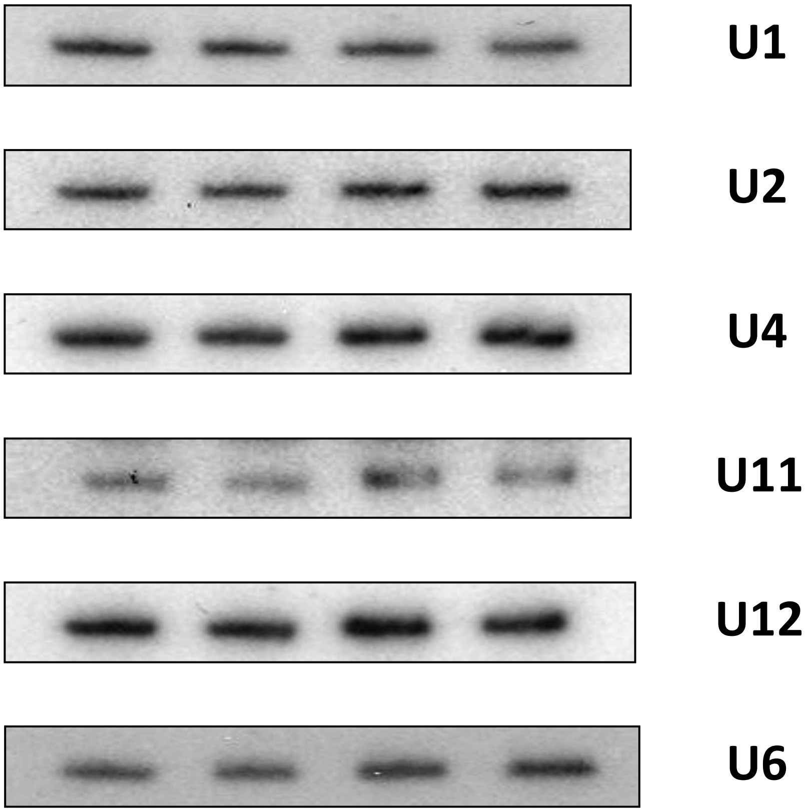

Legend for Fig. S4. Expression of mature U snRNA in patient and control fibroblasts.

Northern blot analysis of the RNA level of different mature U snRNAs in primary fibroblast cultures. From left to right: patient III-4 patient III-2, control 1, control 2.
