# Supplementary material for: Human mutations in integrator complex subunits link transcriptome integrity to brain development
Source: PLoS Genet. 2017 May 25;13(5):e1006809. doi: 10.1371/journal.pgen.1006809 (PMC5466333; doi:10.1371/journal.pgen.1006809)
Supplement: S5 Fig — (PDF) [file pgen.1006809.s006.pdf]

Figure S5

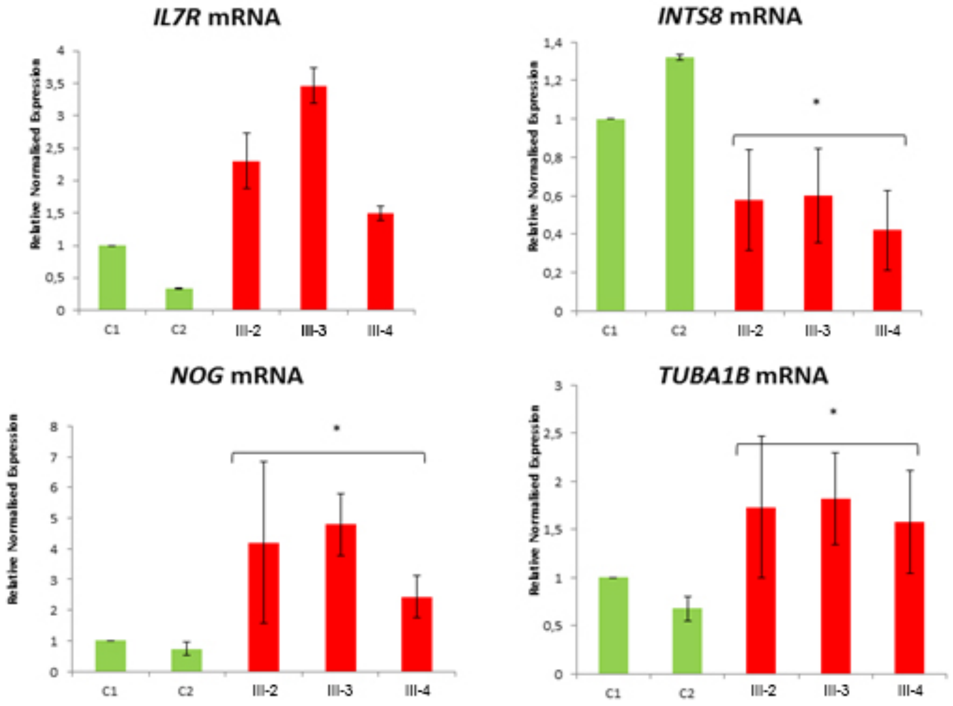

Legend for Fig. S5. qRT-PCR for endogenous *IL7R, TUBA1B* and *NOG* expression: In order to validate reproducibility of the exon arrays results, top upregulated genes from Table S3, expressed in brain and having an important role in development, *TUBA1B* and *NOG* were selected. At the same time, top upregulated gene *ILR7* expressed in brain and detected in the combined list by RNASeq and exon arrays (Table S6) was also selected, tested for qRT-PCR and normalized with housekeeping gene *RNF111* expression (conditions as mentioned in Supplemental Methods). In the same experiment, fibroblasts from the three patients (III-2, III-3, III-4) and two new controls (C1, C2), different from those used for the RNASeq and exon arrays were tested. In the figure, expression for control 1 is used as a refernce and set to 1. Error bars represent +/-SD. Values represent the mean of two independent experiments under identical conditions. *, $P < 0.05$, unpaired Student's t test for three patient sample versus two controls.

Primers used for the three genes are:

TUBA1B_qrt_1F TTCGCCTCCTAATCCCTAGC
TUBA1B_qrt_1R CGTGTTCCAGGCAGTAGAGC

NOG_qrt_1aF CTGGATTCCCATCCAGTACC
NOG_qrt_1aR GGGGATCGATCAAGTGTCC

IL7R_qrt_hu1F GACCCAGATGTCAACATCACC
IL7R_qrt_hu1R CGATGAAATATATCTCTTGTAGTTTCC
