# Supplementary material for: Human mutations in integrator complex subunits link transcriptome integrity to brain development
Source: PLoS Genet. 2017 May 25;13(5):e1006809. doi: 10.1371/journal.pgen.1006809 (PMC5466333; doi:10.1371/journal.pgen.1006809)
Supplement: S6 Fig — (PDF) [file pgen.1006809.s007.pdf]

Figure S6.

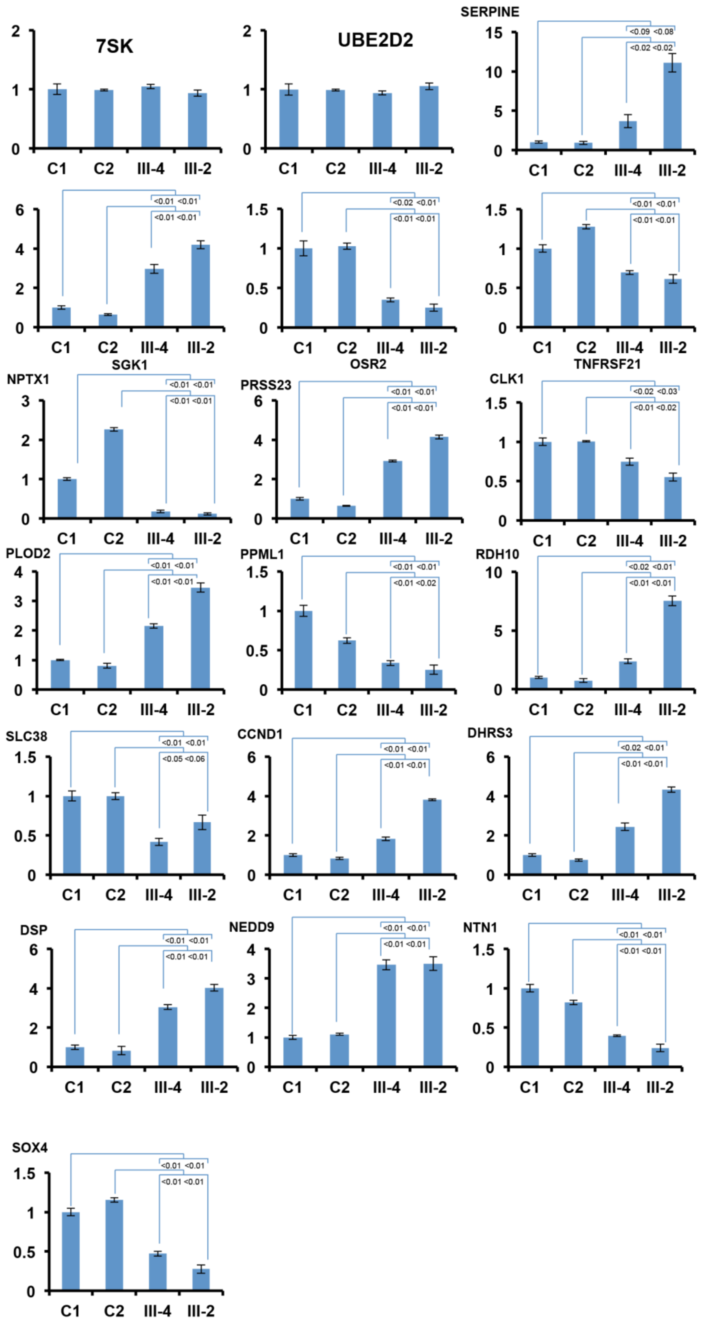

Legend for Fig S6. qRT-PCR validation of differential gene expression in patient fibroblasts, selected from RNA-seq data.

Total RNA from patient (III-2 and III-4) and healthy control (C1 and C2) fibroblasts was extracted, reverse transcribed and analysed by qRT-PCR for the expression of the indicated genes (n=3, +/- SEM).*UBE2D2* expression was used for normalization. See Supplemental Methods for details.
