# Supplementary material for: Human mutations in integrator complex subunits link transcriptome integrity to brain development
Source: PLoS Genet. 2017 May 25;13(5):e1006809. doi: 10.1371/journal.pgen.1006809 (PMC5466333; doi:10.1371/journal.pgen.1006809)
Supplement: S7 Fig — (PDF) [file pgen.1006809.s008.pdf]

Figure S7.

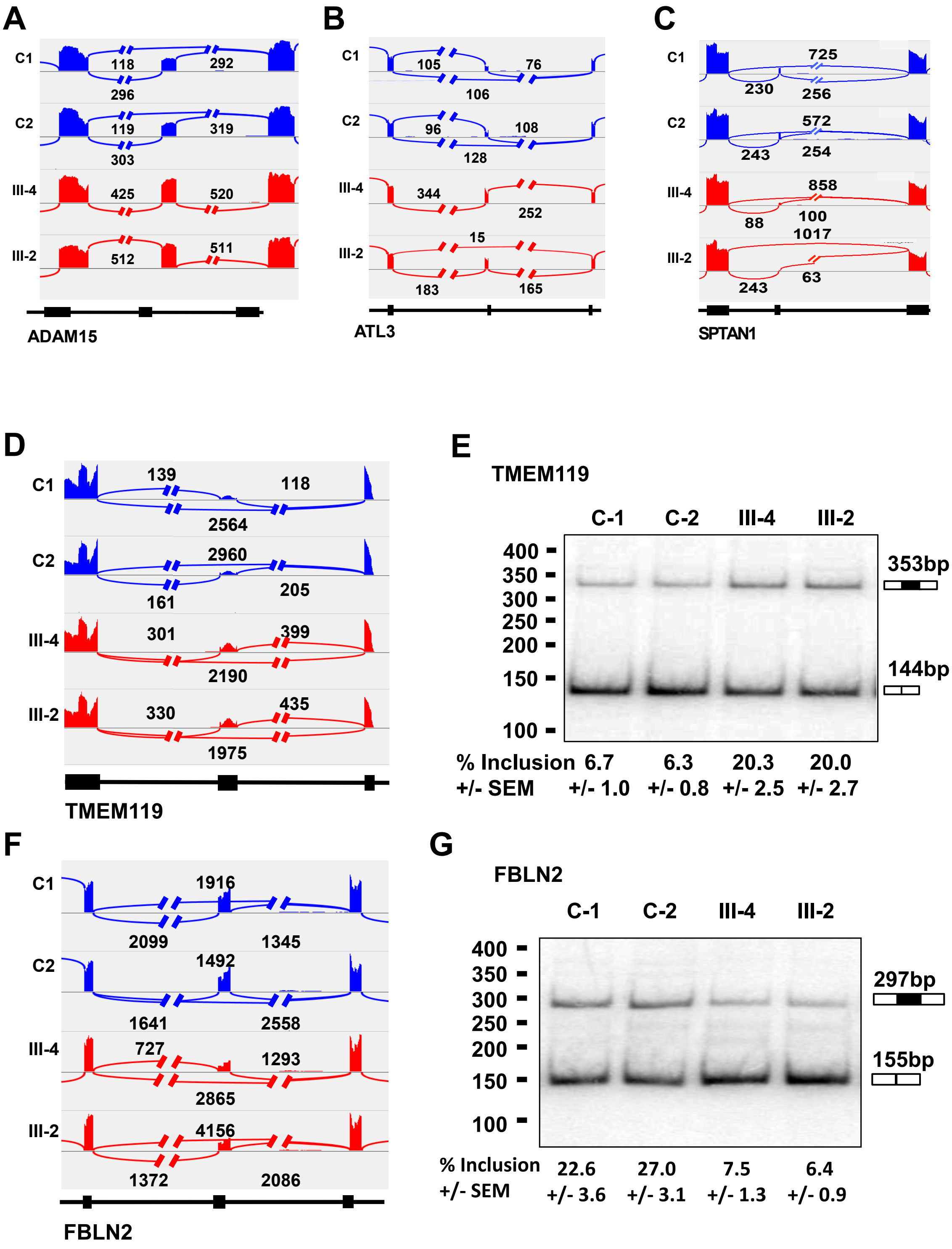
A
C1
118
292
296
C2
119
319
303
III-4
425
520
III-2
512
511
ADAM15
B
C1
105
76
106
C2
96
108
128
III-4
344
252
III-2
15
183
165
ATL3
C
C1
725
230
256
C2
572
243
254
III-4
858
88
100
1017
III-2
243
63
SPTAN1
D
C1
139
118
2564
C2
2960
161
205
III-4
301
399
2190
III-2
330
435
1975
TMEM119
E
TMEM119
C-1
C-2
III-4
III-2
400
350
300
250
200
150
100
353bp
144bp
% Inclusion 6.7 6.3 20.3 20.0
+/- SEM +/- 1.0 +/- 0.8 +/- 2.5 +/- 2.7
F
C1
1916
2099
1345
C2
1492
1641
2558
III-4
727
1293
2865
III-2
4156
1372
2086
FBLN2
G
FBLN2
C-1
C-2
III-4
III-2
400
350
300
250
200
150
100
297bp
155bp
% Inclusion 22.6 27.0 7.5 6.4
+/- SEM +/- 3.6 +/- 3.1 +/- 1.3 +/- 0.9

Legend to Fig S7. Analysis of splicing alterations in patient cells.

A, B, C, D and F. Sashimi plots showing evidence of alternative splicing in the RNAseq data from control (C1 and C2 in blue) and patient (III-2 and III-4 in red) fibroblasts. The number of reads detected for each splicing event is indicated. E and G. Splicing assay for *TMEM119* and *FBLN2*, respectively. Total RNA from control and patient fibroblasts was extracted, reverse transcribed and analysed by PCR using ^{32}P radiolabeled primers to detect alternative splicing. After non-denaturing PAGE and quantification, percentage of exon inclusion was calculated (n=3).
