# Supplementary material for: Human mutations in integrator complex subunits link transcriptome integrity to brain development
Source: PLoS Genet. 2017 May 25;13(5):e1006809. doi: 10.1371/journal.pgen.1006809 (PMC5466333; doi:10.1371/journal.pgen.1006809)
Supplement: S8 Fig — (PDF) [file pgen.1006809.s009.pdf]

**Figure S8.**

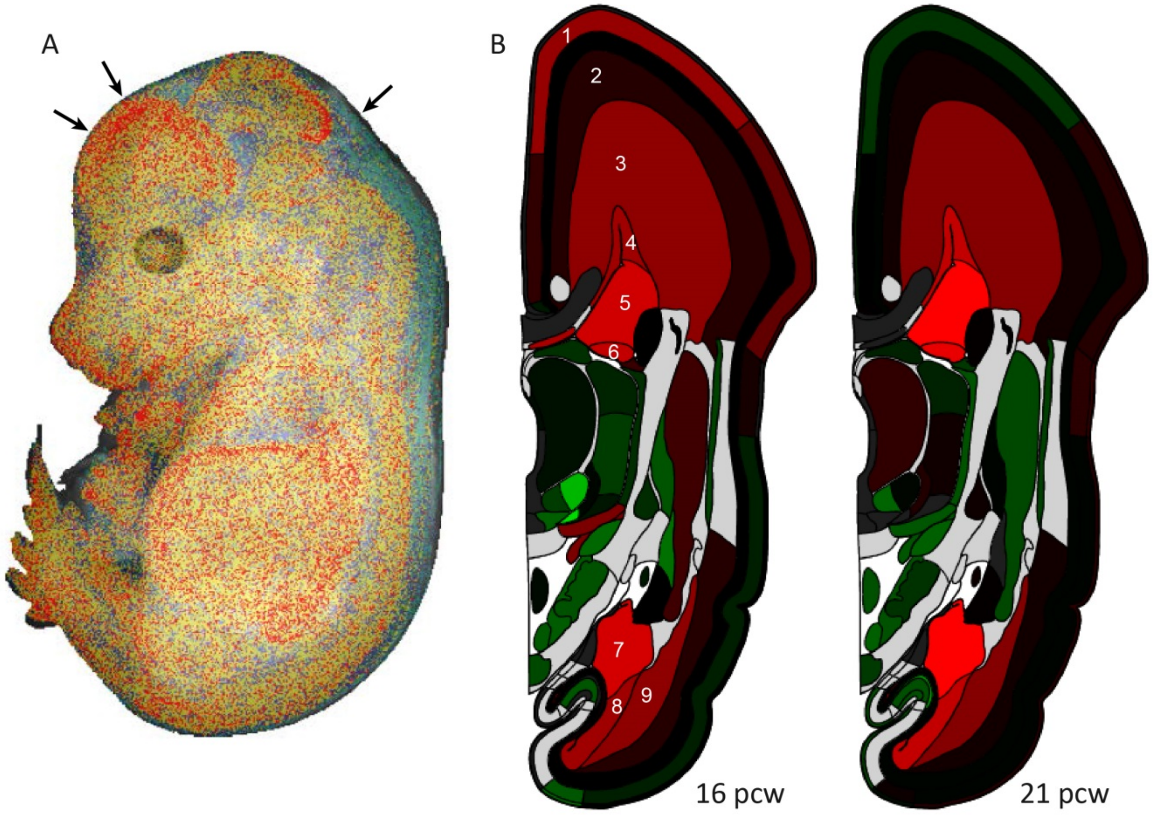


**Legend for Fig. S8. Expression of *INTS8* during fetal development.**

A. Heatmap of *Ints8* RNA FISH in mouse embryonic brain (14.5 dpc) from EMAGE database, showing high expression in brain (red signal), especially in the brain cortex ventricular zone and hindbrain (arrows) (www.emouseatlas.org/gxdb/dbImage/segment2/9884/9884_WM_1.png).(Richardson et al., 2010) B. High expression (red) of *INTS8* in (sub)ventricular zones and ganglionic eminences of the human fetal brain in heatmap of microarray expression data (BrainSpan Atlas of the Developing Human Brain*). 1: cortical plate 2: intermediate zone, 3: subventricular zone (SVZ), 4: ventricular zone (VZ), 5: lateral ganglionic eminences (GE), 6: medial GE, 7: Caudal GE, 8: VZ, 9: SVZ Red indicates above average and green below average brain expression, bright colours differing the most from the mean.

*Allen Institute for Brain Science, BrainSpan Atlas of the Developing Human Brain. 2012 (www.brainspan.org/lcm/gene/34945).
