# Supplementary material for: Human mutations in integrator complex subunits link transcriptome integrity to brain development
Source: PLoS Genet. 2017 May 25;13(5):e1006809. doi: 10.1371/journal.pgen.1006809 (PMC5466333; doi:10.1371/journal.pgen.1006809)
Supplement: S9 Fig — (PDF) [file pgen.1006809.s010.pdf]

Figure S9.

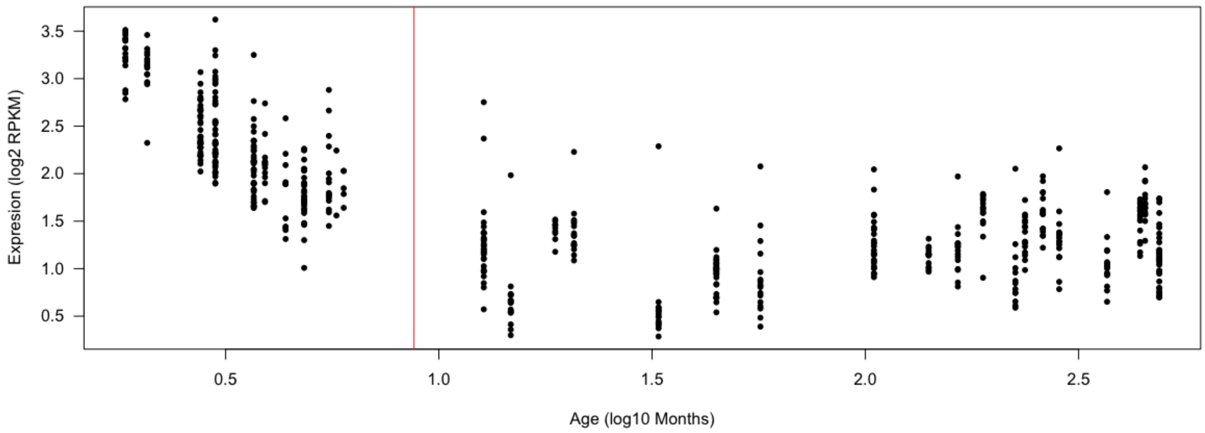

Legend for Fig. S9. RNA sequencing of human brain tissues at several developmental stages.

RNA sequencing data from *INTS8* exon 1-20 at several developmental stages across different human brain tissues (each dot represents the mean of all brain regions for one exon in one individual) were analysed in R and plotted . Y-axis represents the *INTS8* RNA expression, X-axis represents the age of the subject (range 8 pcw - 40 years). The vertical red line separates prenatal from postnatal data. Raw data were obtained from **BrainSpan Atlas of the Developing Human Brain. (http://www.brainspan.org/rnaseq/gene/1098436).**
