# Supplementary material for: Human mutations in integrator complex subunits link transcriptome integrity to brain development
Source: PLoS Genet. 2017 May 25;13(5):e1006809. doi: 10.1371/journal.pgen.1006809 (PMC5466333; doi:10.1371/journal.pgen.1006809)
Supplement: S10 Fig — (PDF) [file pgen.1006809.s011.pdf]

Figure S10.

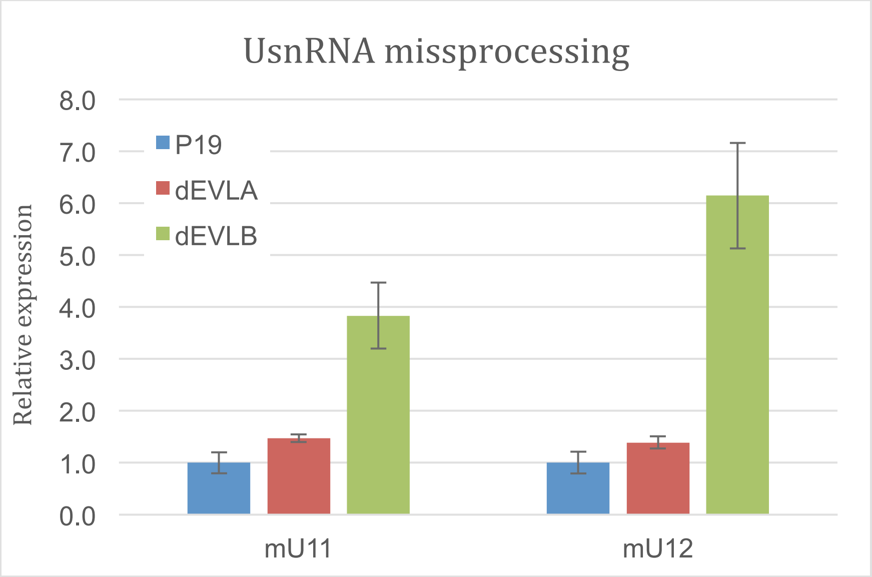

Legend for Fig. S10.

qRT-PCR showing normalized expression of misprocessed U11, U12 in total RNA extracted from wild-type and mutant P19 cell line, data is normalize to 5S RNA expression. All pairwise comparisons between wild type and mutant UsnRNA levels are significant (n=3, $p<0.05$, Student's T-test).

Primers used:

5S_F	TACGGCCATACCACCCTGAA
5S_R	GCGGTCTCCCATCCAAGTAC
mU11_F	TTGGTAATTGGGCAGCTGGT
mU11_R	AAGCTTCCTCCTGTAATTTAACAAA
mU12_F	TACTTTGCGGGATGCCTGG
mU12_R	ACTAGCACCTGAGAGCAACC
