# Supplementary material for: Human mutations in integrator complex subunits link transcriptome integrity to brain development
Source: PLoS Genet. 2017 May 25;13(5):e1006809. doi: 10.1371/journal.pgen.1006809 (PMC5466333; doi:10.1371/journal.pgen.1006809)
Supplement: S1 Table — (DOCX) [file pgen.1006809.s012.docx]

**Table S1: Whole genome sequencing (WGS)**
