# Supplementary material for: Human mutations in integrator complex subunits link transcriptome integrity to brain development
Source: PLoS Genet. 2017 May 25;13(5):e1006809. doi: 10.1371/journal.pgen.1006809 (PMC5466333; doi:10.1371/journal.pgen.1006809)
Supplement: S3 Table — (DOCX) [file pgen.1006809.s014.docx]

**Table S3: Results of *in silico* predictions of *INTS8* mutation effect and conservation across species by prediction software**

| \| **software** \|  \| **p.Asp298Gly** \|  \|  \|  \|  \| **p.Glu973_Leu975del** \| \| \| \| --- \| --- \| --- \| --- \| --- \| --- \| --- \| --- \| --- \| --- \| \| SIFT \|  \| damaging, 0.046 \| \|  \|  \|  \| NA \|  \|  \| \| PROVEAN \| \| deleterious, -3.507 \| \|  \|  \|  \| deleterious, -8.989 \| \|  \| \| Polyphen2 \| \| probably damaging, score 0.998 \| \| \| \|  \| NA \|  \|  \| \| mutation accessor \| \| medium, 2.045 \| \|  \|  \|  \| NA \|  \|  \| \| mutation taster \| \| disease causing, prob: 0.999999977310485 \| \| \| \| \| disease causing, prob: 0.533443002797038 \| \| \| \| FATHMM \|  \| damaging, -3.48 \| \|  \|  \|  \| NA \|  \|  \| \| SNAP \|  \| non-neutral, expected accuracy 58% \| \| \| \|  \| NA \|  \|  \| \| CADD(PHRED) \|  \| 28.8 \| \| \| \|  \| 19.34 \|  \|  \| |  |  |  |  |  |  |  |  | | |  |
| --- | --- | --- | --- | --- | --- | --- | --- | --- | --- | --- | --- | --- | --- | --- | --- | --- | --- | --- | --- | --- | --- | --- | --- | --- | --- | --- | --- | --- | --- | --- | --- | --- | --- | --- | --- | --- | --- | --- | --- | --- | --- | --- | --- | --- | --- | --- | --- | --- | --- | --- | --- | --- | --- | --- | --- | --- | --- | --- | --- | --- | --- | --- | --- | --- | --- | --- | --- | --- | --- | --- | --- | --- | --- | --- | --- | --- | --- | --- | --- | --- | --- | --- | --- | --- | --- | --- | --- | --- | --- | --- | --- | --- | --- | --- | --- | --- | --- | --- | --- | --- | --- |
| NA= not assessed, the p.Glu973_Leu975del could not be assessed in all software being a deletion. | |  | | | | |  |  |  |  |  |
|  | |  | |  |  |  |  |  |  |  |  |
| 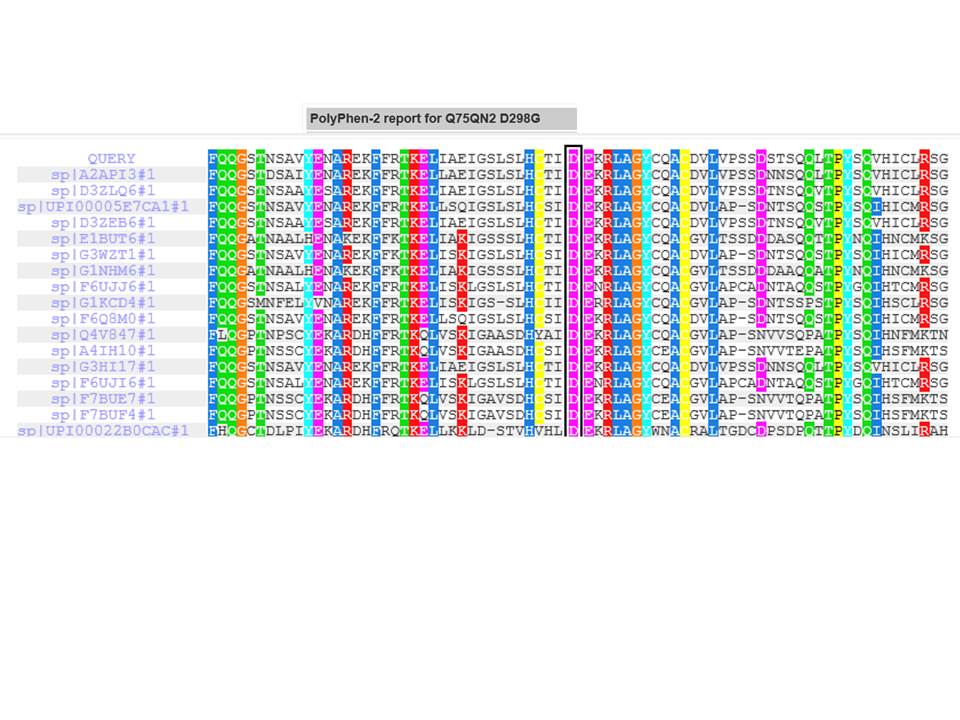 | |  | | | | |  |  | | | |

**
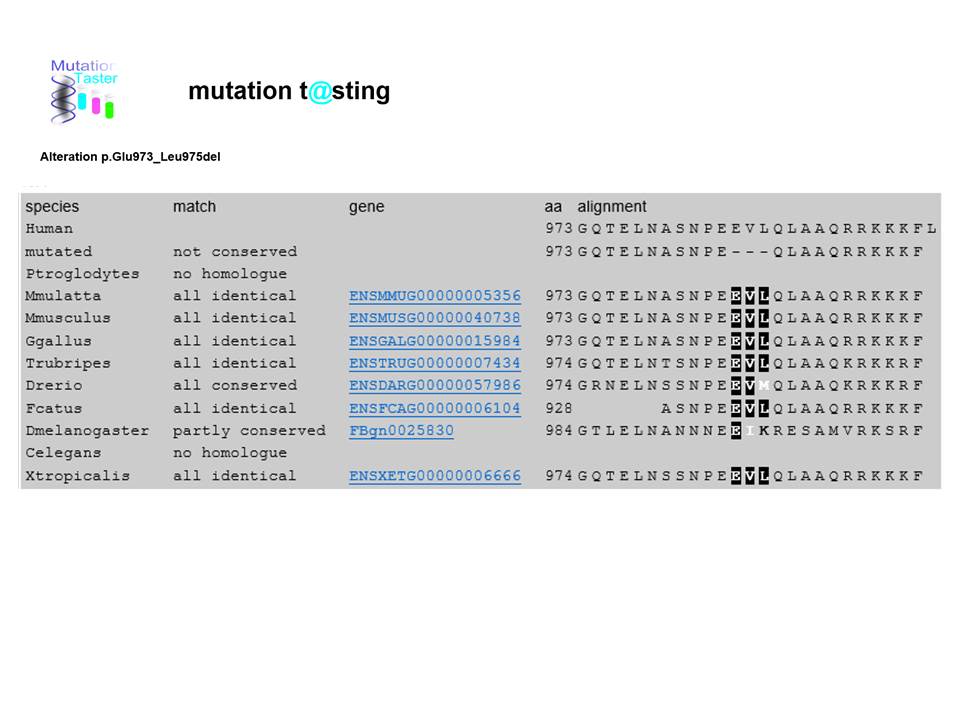
**
